# The Role and Mechanism of Carnosine in Alleviating Type 2 Diabetic Sarcopenia in Mice Through PI3K/AMPK/PGC-1α Signaling Pathway

**DOI:** 10.3390/biology15130999

**Published:** 2026-06-25

**Authors:** Xiang Li, Bo Tian, Yuxin Chen, Huili Tong, Xiaoming Chen, Zhifeng Cheng

**Affiliations:** 1 Department of General Practice and Department of Endocrinology and Metabolism, The Fourth Affiliated Hospital of Harbin Medical University, Harbin 150001, China; 840307@hrbmu.edu.cn (X.L.);; 2 Key Laboratory of Animal Cellular and Genetics Engineering of Heilongjiang Province, Northeast Agricultural University, Harbin 150030, China

**Keywords:** carnosine, mice, T2DM, sarcopenia, C2C12 myoblasts

## Abstract

Type 2 diabetes mellitus (T2DM) accounts for over 90% of diabetes cases, and T2DM-related sarcopenia is a growing concern. Carnosine, abundant in human skeletal muscle, helps maintain muscle quality and function. This study investigated whether carnosine deficiency contributes to T2DM-related sarcopenia and whether exogenous carnosine supplementation alleviates muscle atrophy. A mouse model of T2DM sarcopenia was established using streptozotocin combined with a high-fat diet. LC-MS metabolomics revealed a significant reduction in carnosine content in the gastrocnemius muscle of model mice. A C2C12 myotube atrophy model was induced by high-glucose (HG), and qRT-PCR showed altered expression of carnosine metabolism-related enzymes, suggesting disrupted carnosine homeostasis under T2DM conditions. Mechanistic investigations using immunofluorescence, Western blotting, transcriptome sequencing, mitochondrial staining, and molecular docking indicated that carnosine may alleviate high-glucose-induced myotube atrophy through the PI3K/AMPK/PGC-1α signaling pathway. In vivo, carnosine supplementation increased the number of mitochondria and the proportion of slow muscle fibers in gastrocnemius muscle, ameliorating the atrophic phenotype. These findings suggest that carnosine has potential as a candidate for intervention in T2DM-related sarcopenia, though further validation of its direct molecular targets is required.

## Simple Summary

Type 2 diabetes (T2DM) often leads to skeletal muscle loss and weakness, a condition called sarcopenia, which worsens human health. This study explored whether carnosine, a natural dipeptide found in meat and present in human muscle, can help protect against muscle wasting. Using a T2DM sarcopenia mouse model and a high-glucose-treated C2C12 myotube in vitro model, the researchers found that carnosine levels were reduced in T2DM sarcopenia muscles. Oral carnosine supplementation in the mice helped alleviate skeletal muscle atrophy. Carnosine works by activating a cellular signaling pathway that boosts the number of mitochondria (the energy powerhouses of cells) and shifts muscle fibers toward a more oxidative type, leading to stronger and healthier muscles in T2DM sarcopenia mice. These findings suggest that carnosine supplementation could be a simple and promising nutritional strategy to help manage skeletal muscle loss in people with type 2 diabetes, though further studies in humans are needed to confirm its effectiveness.

## 1. Introduction

Diabetes is a serious global public health challenge. Population aging and lifestyle changes have continued to increase the prevalence of diabetes. Diabetes is characterized by hyperglycemia, and its pathophysiology involves dysfunction of multiple organs, such as β-cells and adipose tissue. According to the WHO (2019), diabetes is classified into T1DM (caused by β-cell immune destruction), T2DM (related to insulin resistance), and other subtypes, with T2DM accounting for the vast majority of cases [1]. T2DM is a major global public health challenge, currently affecting over 537 million adults, with projections reaching 783 million by 2045 [2]. Long-term hyperglycemia leads to multi-organ complications. As a new complication, diabetic sarcopenia has become a research hotspot in metabolic diseases [3].

Diabetic sarcopenia is an age-related metabolic disease featuring progressive loss of muscle mass, decreased muscle strength, and motor dysfunction, often accompanied by poor blood glucose control [4]. The incidence of sarcopenia in patients with T2DM is significantly higher than that in the normal population. Skeletal muscle decline occurs in the early stages of the disease, and it is more obvious with age, forming a vicious cycle with T2DM [5]. It is caused by the interaction of multiple factors, such as metabolic disorders and chronic inflammation [6]. Diabetes can accelerate skeletal muscle damage and aggravate blood glucose disorders through lipotoxicity, oxidative stress, and other factors [7]. Skeletal muscle is the largest metabolic organ in the human body (about 40% of body weight) and plays a key role in metabolic regulation. Its functional decline increases the mortality, disability, and fall risk of patients, and increases the social and economic burden [8,9,10]. Therefore, it is of great theoretical and clinical value to explore the intervention targets and mechanisms of T2DM-related sarcopenia.

Although there are many drug treatments for diabetes, there are no drugs approved for the treatment of sarcopenia, so the treatment of sarcopenia is mainly non-drug intervention. Due to the complex pathophysiological mechanisms of T2DM sarcopenia, patients have significant individual differences in age, gender, and physiological status, and their intervention and treatment need to be targeted. Nutritional intervention and exercise are both important methods of non-drug intervention.

Carnosine is a natural dipeptide composed of β-alanine and L-histidine, first discovered in meat extracts more than 100 years ago. Carnosine is abundant in mammalian skeletal muscle, myocardium, and brain. Dietary carnosine is mostly decomposed into β-alanine and L-histidine after digestion, and the undigested portion is absorbed into the blood via the PEPT1 transporter on intestinal epithelial cells. Serum carnosinase CN1 (EC 3.4.13.20) rapidly degrades carnosine, making its blood concentration unstable. Skeletal muscle efficiently takes up and enriches carnosine through PEPT2 and CA3. At the same time, skeletal muscle cells uptake precursor amino acids through membrane-specific transporters (PAT1 and TauT for β-alanine, PHT1 for L-histidine) and synthesize carnosine via carnosine synthase (CARNS). The cytoplasmic carnosinase CN2 (EC 3.4.13.18), encoded by the *CNDP2* gene, degrades carnosine and maintains its dynamic balance [11,12].

Clinical studies have shown that daily supplementation of 2 g of carnosine for 12 weeks can improve insulin resistance in patients with T2DM [13]. Combined with anti-diabetic drugs, it can also reduce blood glucose, triglyceride, and TNF-α levels in patients [14], suggesting its potential value in the prevention and treatment of T2DM complications. Carnosine exhibits antioxidant, anti-inflammatory, anti-glycation, and mitochondrial protective activities. It may alleviate T2DM sarcopenia through multiple mechanisms. First, it scavenges ROS produced by high skeletal muscle metabolism, thereby reducing oxidative stress [15,16]. Second, it inhibits the formation of AGEs (Advanced Glycation End Products), which alleviates subsequent inflammation and oxidative stress [17]. Third, it suppresses pro-inflammatory cytokines, leading to reduced skeletal muscle protein breakdown and enhanced protein synthesis [18]. Fourth, it stimulates coenzyme Q10 biosynthesis to improve mitochondrial function, counteracting diabetes-induced energy deficits and skeletal muscle injury [19]. However, the precise molecular mechanisms by which carnosine alleviates T2DM sarcopenia remain unclear and require in-depth exploration.

In this study, LC-MS metabolomics sequencing was used to analyze the skeletal muscle tissue of T2DM sarcopenia mice, and significantly altered metabolites related to T2DM sarcopenia were identified. It was found that the content of carnosine in skeletal muscle of model mice was significantly reduced, suggesting that the decrease in carnosine levels may be closely related to the pathological process of T2DM sarcopenia. Exogenous carnosine supplementation significantly ameliorated skeletal muscle atrophy in vivo. Furthermore, using a high-glucose-induced C2C12 myotube atrophy model combined with high-throughput transcriptome sequencing and molecular biology assays, we systematically elucidated that carnosine promotes mitochondrial biosynthesis through PI3K/AMPK/PGC-1α signaling pathway. Together, these findings provide new insights into the mechanism of carnosine in T2DM sarcopenia and lay a foundation for its potential clinical translation.

## 2. Materials and Methods

### 2.1. Experimental Materials

Animals: Wild-type 4-week-old male ICR mice were purchased from Liaoning Changsheng Biology and raised in the Experimental Animal Center of Northeast Agricultural University. All animal experiments were approved by the Animal Protection Committee of Northeast Agricultural University (Animal Ethics No.: NEAUEC20250119, the approval date of the protocol is 12 March 2025), and carried out in accordance with the “Chinese Animal Science Standards” of the National Standardization Technical Committee of Animal Science. All the experimental operators had the qualification certificate of experimental animal employment training issued by Heilongjiang Province. Feeding conditions: a 12 h light/12 h dark circadian rhythm cycle, constant temperature and humidity, sufficient food and water supply. The mice were acclimatized for 1 week before the experiment.

Cell line: The mouse C2C12 myoblast cell line was purchased from Procell System (Procell, Wuhan, China), No.: CL-0040. C2C12 Cell Complete Medium (C2C12 special medium) containing 10% fetal bovine serum was used for culture. Culture conditions: constant temperature and humidity (37 °C), 5% CO_2_ concentration. When the cells were confluent at 60–70%, they were seeded into cell bottles or well plates to induce differentiation. After differentiation into myotubes, high-glucose was added to the culture medium, and the myotube atrophy experiment was used to simulate the high-glucose environment of skeletal muscle in vivo so as to study the molecular mechanism.

### 2.2. Reagents

Streptozotocin (STZ, S0130) was purchased from Sigma-Aldrich (Shanghai, China). L-Carnosine (G2216140) was obtained from Aladdin Reagent Co., Ltd. (Shanghai, China). Fetal bovine serum (FBS, 04-001-1ACS) and horse serum (04-124-1A) were acquired from Biological Industries (Shanghai, China). Penicillin–streptomycin solution (100×, S17032), trypsin, Triton X-100 (R21239), and LY294002 (S43088) were sourced from Yuanye Bio-Technology (Shanghai, China).

For quantitative real-time PCR (qRT-PCR) analysis, PowerTrack SYBR Green Master Mix (A46109) was purchased from Thermo Scientific (Waltham, MA, USA). The SPARKeasy Total RNA Fast Extraction Kit (AC0202) and SPARKscript II 1st Strand cDNA Synthesis Kit (AG0302) were obtained from Sparkjade Biotechnology (Jinan, Shandong, China). All primer sequences used for SYBR Green-based qRT-PCR are listed in Table S1.

For Western blotting analysis, phenylmethylsulfonyl fluoride (PMSF, ST505), RIPA lysis buffer (P0013B), SDS-PAGE protein loading buffer (P0015), prestained protein marker (P0068), and the SDS-PAGE Gel Fast Preparation Kit (stain-free, P0925S) were purchased from Beyotime Biotechnology (Shanghai, China). PVDF transfer membranes (0.45 μm, 88518) were obtained from Thermo Scientific (Shanghai, China). Enhanced chemiluminescence (ECL) substrate (cat. no. AR1027) was acquired from Bioster (Wuhan, China). Details of all antibodies used in this study are provided in Table S2.

For histological staining, paraformaldehyde (441244), paraffin wax (P3558), and Tween-20 (P9416) were purchased from Sigma-Aldrich (Shanghai, China). Hematoxylin (R20568), eosin (R24045), and DAPI nuclear staining solution (R20276) were sourced from Yuanye Bio-Technology (Shanghai, China). Citrate antigen retrieval solution (P0081) and antifade mounting medium (P0218S) were obtained from Beyotime Biotechnology. Neutral balsam (BL704A) was purchased from Biosharp (Shanghai, China).

### 2.3. Establishment of T2DM Sarcopenia Mouse Model

Four-week-old male ICR mice (initial body weight approximately 16–20 g) were used in this study to avoid hormonal interference. All mice were housed under standard conditions (12 h light/dark cycle, constant temperature and humidity, free access to food and water). The mice were randomly divided into three groups: control group (normal chow), T2DM sarcopenia group (high-fat diet + STZ), and carnosine treatment group (high-fat diet + STZ + carnosine supplementation). Each group contained six mice (*n* = 6). The in vivo experiment was designed with randomization for group allocation and blinding for outcome assessment. All animal procedures were performed under anesthesia induced by intraperitoneal injection of Avertin (tribromoethanol, 250 mg/kg, Dowobio Biotechnology Co., Ltd., Shanghai, China). The mice were euthanized by cervical dislocation under deep anesthesia at the end of the study. The animals were monitored daily for signs of pain or distress, and no adverse events were observed.

For T2DM induction, mice in the experimental groups were first fed a high-fat diet (D12492, Xiao Shu You Tai Biotechnology, Beijing, China) for 1 month. Subsequently, they received intraperitoneal injections of streptozotocin (STZ, S0130, Sigma-Aldrich, Shanghai, China) at a dose of 50 mg/kg body weight once daily for 5 consecutive days. STZ was freshly dissolved in 0.1 M citrate buffer (pH 4.5) and used immediately. The control group received an equivalent volume of citrate buffer.

Blood glucose levels were measured from tail vein blood 3–7 days after the last STZ injection. Mice with fasting blood glucose ≥ 11.1 mmol/L on two consecutive measurements were considered successfully modeled for T2DM. After T2DM confirmation, the mice were maintained under the same diet conditions for an additional 3 months to allow for the development of sarcopenia. Throughout the entire experimental period (1 month of high-fat diet, 5 days of STZ injection, followed by 3 months of maintenance), body weight and blood glucose were monitored regularly. After the T2DM sarcopenia mouse model was successfully established, four mice from each group were randomly selected, and their gastrocnemius muscles were collected for subsequent metabolomic sequencing.

At the end of the maintenance period, forelimb grip strength was measured using a commercial grip strength meter (Model LLY, Shanghai Puxin Instrument Technology Co., Ltd., Shanghai, China) to assess muscle function. Each mouse was lowered over a metal grid, allowing only its forepaws to grip. The experimenter then gently pulled the mouse backward by its tail, and the peak force (in grams) recorded by the force sensor was taken as the grip strength. The test was repeated 10 times for each mouse, and the average value was calculated. All measurements were performed at the same time of day to control for circadian influences.

The mice were then euthanized, and gastrocnemius muscles were dissected, weighed, and processed for histological and biochemical analyses. Skeletal muscle atrophy was evaluated by muscle wet weight ratio (muscle weight/body weight), hematoxylin and eosin (HE) staining, and measurement of muscle fiber diameter and cross-sectional area (CSA).

For carnosine intervention, the carnosine + T2DM sarcopenia group received oral administration of carnosine (1 g/L in drinking water) for 30 consecutive days after T2DM was confirmed. The control and T2DM sarcopenia groups received normal drinking water.

### 2.4. Metabolomic Sequencing of Skeletal Muscle Tissue in T2DM Sarcopenia Mice

The mouse skeletal muscle tissue was placed in a tissue grinding tube, 200 μL RIPA (containing protease inhibitor) was added, and the tissue was broken with a tissue homogenizer and placed on ice for 30 min. After the cells and tissue were fully lysed, they were centrifuged at 4 °C with a low-temperature centrifuge at 12,000 rpm for 10 min, and the supernatant was taken. The absorbance of the standard and experimental samples at 562 nm was measured on a microplate reader (Agilent Technologies, Santa Clara, CA, USA). The concentration of the sample was calculated according to the absorbance curve of the standard protein sample provided by the Pierce BCA kit (Thermo Scientific, Waltham, MA, USA).

Non-targeted LC-MS metabolomics was performed by Majorbio (Shanghai, China). Gastrocnemius muscle samples (*n* = 4 per group) were analyzed by Majorbio (Shanghai, China) using a Vanquish UHPLC system coupled to a Q-Exactive Orbitrap mass spectrometer (Thermo Fisher Scientific). Separation was performed on an HSS T3 column (2.1 × 100 mm, 1.8 μm) at 40 °C with a water–acetonitrile gradient (0.1% formic acid). Data were acquired in both positive and negative ESI modes (*m*/*z* 70–1050). Pooled QC samples were injected every 10 runs; metabolites with QC RSD >30% were excluded. Raw data were processed with Progenesis QI, and metabolites were identified against mzCloud, HMDB, and KEGG databases (mass tolerance ≤ 5 ppm, match score ≥ 70%). Total peak area normalization was applied. Differential metabolites were defined as |log_2_FC| ≥ 1 and *p* < 0.05 (Student’s *t*-test).

### 2.5. Paraffin Embedding and Slicing of Skeletal Muscle Tissue

Fresh skeletal muscle tissue was fixed in 4% paraformaldehyde prepared with PBS (pH 7.4), incubated in a shaker for 16 h (changing the fixative once), and washed twice with distilled water after discarding the fixative. The fascia and fat were removed under an anatomical microscope, and the cuboid (section perpendicular to the muscle axis) was repaired along the muscle fibers. The tissues were dehydrated with 30–100%-grade ethanol series (with each concentration applied twice for 30 min), and cleared in xylene twice (30 min each) until translucent (amber); the paraffin was preheated at 65 °C, and the tissue was immersed in paraffin twice (1 h each). Pure paraffin was injected into the embedding box to remove bubbles, and the labeled samples were placed vertically to be solidified. Then, the wax block was removed and the slices were unfolded in a water bath at 42 °C for 15 s, dried at 37 °C for 2 h, baked overnight at 60 °C, cooled at room temperature for 30 min, and then transferred to a slice box for drying and preservation.

### 2.6. Hematoxylin & Eosin (HE) Staining

Tissue sections (7 μm) were baked at 60 °C to remove residual moisture, dewaxed in fresh xylene twice (10 min each with gentle agitation), and rehydrated through graded ethanol series: absolute ethanol twice (2 min each), as well as 95%, 85%, and 75% ethanol (2 min each), followed by a brief rinse in PBS (10 s) on a horizontal shaker (60 rpm). For hematoxylin and eosin (H&E) staining, sections were stained with hematoxylin (pre-warmed to 37 °C) for 3 min, rinsed with distilled water for 10 s, differentiated in 0.5% acid alcohol (75% ethanol) until the tissue appeared light purple under microscopic examination, and washed for 20 s. The sections were then counterstained sequentially with 0.25% eosin (containing 0.2% calcium chloride) for 5 s and 0.5% eosin for 30 s, followed by a rinse in distilled water for 10 s. Subsequently, sections were dehydrated through ascending ethanol series (70%, 80%, 90%, 95%, and 100% × 2; 5–10 s each step, with excess liquid removed between steps), cleared in xylene twice (1 min each), and mounted with pre-warmed neutral balsam (45 °C). The slides were dried at 37 °C for 12 h and stored at 4 °C in the dark.

### 2.7. Immunofluorescence Staining of Tissue Sections

After baking at 60 °C for 30 min, the tissue sections were digested with 0.1% protease K-Tris-HCl (pH 8.0) at 37 °C for 5 min to complete antigen repair and reduce non-specific adsorption. The slices were deparaffinized by xylene 2 times, absolute ethanol 2 times, 90–50% ethanol 1 time, and distilled water 2 times, each time for 5 min. Then, 5% BSA-PBST blocking solution was added (PBS preparation: 0.01 M pH 7.4, containing 8 g NaCl, 0.2 g KCl, 2.9 g Na_2_HPO_3_·12H_2_O, 0.2 g KH_2_PO_4_, with pure water added to 1 L; PBST preparation: 1 L PBS and 1 mL Triton X-100 magnetic stirring for 10 min; preparation of blocking solution: 5 g BSA and 100 mL PBST), and the samples were kept in a 37 °C wet box for 30 min to reduce the background. Then, the blocking solution was discarded, and the primary antibody diluted in 5% BSA-PBST was added dropwise and placed in a 50% glycerol–PBS wet box at 4 °C overnight. After the primary antibody was recovered, the samples were washed three times with PBST (10 min each time), the secondary antibody was diluted in 5% BSA-PBST and added dropwise, and the tissue sections were allowed to stand at 37 °C for 1 h. After the second antibody was discarded, the samples were washed three times with PBST (10 min each time), DAPI was added and left to stand at room temperature for 10 min, the samples were washed three times again with PBST (5 min each time), and the anti-fluorescence quencher was observed and photographed. If double or triple staining was performed, serum blocking was repeated 1–2 times up to the second antibody incubation step before nuclear staining.

### 2.8. Determination of Carnosine Concentrations by ELISA

The mice were anesthetized, and blood was collected via retro-orbital bleeding. Plasma was separated by centrifugation at 3000 rpm for 10 min at 4 °C and stored at −80 °C. Additionally, whole blood was collected via ocular puncture (or orbital extraction) and processed to obtain serum. Bilateral gastrocnemius muscles were dissected and homogenized in ice-cold PBS (1:9, *w*/*v*). Homogenates were centrifuged at 10,000 rpm for 15 min at 4 °C, and supernatants were collected and stored at −80 °C. Carnosine concentrations in serum and muscle lysates were quantified using a Mouse Carnosine ELISA Kit (YJ742901, YunanjuBio, Shanghai, China) according to the manufacturer’s instructions.

### 2.9. Subculture of C2C12 Myoblasts

Regular spindle-shaped cells were selected with a confluence of 70–80% (avoiding ≥ 90% to prevent high-density induction of early differentiation or flaking off); the cell culture bottle was taken out, the culture medium was discarded, and 2 mL PBS containing double antibiotics was added along the side wall to gently shake and wash all the cells. One mL of 0.25% trypsin containing EDTA was added, and the cells were placed in an incubator at 37 °C for 45–60 s. When 70% of the cells became round and did not fall off, 3 mL of complete medium (DMEM + 10% FBS + 1% P/S) was added to terminate digestion (avoiding excessive digestion and reducing differentiation potential). After the cell suspension was blown and mixed, it was inoculated into a new culture flask or a well plate according to the experimental requirements. The cells were shaken horizontally to ensure that the cells adhered to the wall evenly and cultured in an incubator. The cell growth status was observed after 24 h.

### 2.10. Establishment of an In Vitro Myotube Atrophy Model in Mice

Differentiation of C2C12 myoblasts: Upon reaching 60–70% confluence, the proliferation medium was aspirated and replaced with differentiation medium to induce myogenic differentiation.

After 3 days of differentiation, the formation of multi-nucleated myotubes was confirmed by microscopy, indicating that the myotubes were fully differentiated before high-glucose treatment. The differentiation medium was refreshed periodically during this interval, with changes performed as needed based on observed medium color shifts (indicating pH changes). The differentiation medium (100 mL) was prepared as follows: 97 mL basal medium (DMEM) supplemented with 2 mL horse serum and 1 mL 100× penicillin–streptomycin solution.

High-glucose treatment to induce myotube atrophy: After the successful fusion of C2C12 cells into mature myotubes, the differentiation medium was removed. Experimental groups were incubated with 2 mL of serum-free DMEM containing 10 mM D-glucose per well, and maintained at 37 °C in a humidified atmosphere with 5% CO_2_ for 24 h to induce atrophic conditions.

### 2.11. Immunofluorescence Staining of Myotubes

C2C12 myoblasts were induced to differentiate by 2% horse serum for 3 days, and stained when ≥80% of the cells formed multinucleated myotubes under the microscope. After the culture medium was discarded, the cells were washed once with PBS, and 1 mL 4% paraformaldehyde was added for fixation for 10 min at room temperature. After the fixation solution was discarded, the cells were incubated with PBS containing 0.1% Triton X-100 for 10 min to penetrate the membrane (eliminating repeated washing to reduce myotube shedding). Subsequently, 5% BSA PBS blocking solution was added and the cells were incubated at 37 °C for 1 h. After discarding the blocking solution, Anti-Desmin primary antibody (1:200, 5% BSA PBS dilution) was added and the cells were incubated at 4 °C in a wet box overnight. After washing the cells with PBST 3 times (10 min each time), the cells were incubated with fluorescent secondary antibody diluted with blocking solution (1:100) in a wet box at 37 °C for 1 h in the dark. After the second antibody incubation, PBST was used to wash the membrane three times (10 min each time), 500 μL DAPI was added and the cells were placed at room temperature for 5 min. PBST was used to wash the cells three times, and an anti-fluorescence quencher was added to observe and take pictures. Quantitative analysis was performed using ImageJ 1.54u2. Myotube fusion rate = (number of nuclei containing ≥ 2 nuclei)/total number of nuclei × 100% (5 random fields per well, total number of nuclei ≥ 500). After 8-bit conversion, automatic thresholding, and skeletonization of Desmin fluorescence images, Feret diameter ≥ 150 per 30 μm was measured vertically.

### 2.12. Western Blotting

C2C12 cells or skeletal muscle tissue samples were homogenized with 400 μL RIPA solution before loading. The supernatant was centrifuged at 12,000 rpm for 10 min at 4 °C, and 100 μL 5 × loading buffer was added to a boiling water bath for 10 min. The protein concentration was calculated by BCA assay for electrophoresis. The separation gel and the concentrated gel were configured according to the kit instructions, and the pre-dyed marker was added when the sample was loaded. The electrophoresis was performed at 80 V for 90 min and then increased to 120 V for 30 min. After electrophoresis, the transfer printing clip was assembled in the order of sponge pad–filter paper–glue–PVDF membrane–filter paper–sponge pad (PVDF membrane was activated with methanol), and the membrane was transferred to an ice bath at 200 mA for 90 min. After the membrane was transferred, it was incubated with 5% skimmed milk PBST blocking solution at 37 °C for 1 h, the primary antibody was incubated overnight at 4 °C, the sample was washed with PBST three times (10 min each time), the secondary antibody was incubated at 37 °C for 1 h, the sample was washed with PBST again three times, and ECL luminescent solution was added dropwise to expose the image.

### 2.13. Transcriptome Sequencing

Total RNA was extracted from myotubes treated with high-glucose, carnosine and high-glucose. RNA integrity was analyzed by gel electrophoresis. The RNA concentration and OD260/OD280 value were measured by NanoDrop 2000C, and samples with 260/280 = 1.9–2.1 and 260/230 ≥ 2.0 can be used for subsequent experiments. Library construction: The library was constructed according to the total mRNA sequence. Sequencing: Library construction was performed using the KAITAI U-mRNAseq Library Prep Kit (AT4221) following the manufacturer’s protocol. Sequencing was carried out on an Illumina NovaSeq 6000 platform in paired-end 150 bp (PE150) mode, generating approximately 6 Gb of clean data per sample. Raw reads were processed using fastp (v0.23.4) to remove adapter sequences and low-quality reads. Clean reads were aligned to the mouse reference genome (GRCm38/mm10, Ensembl) using HISAT2 (v2.2.1); the average mapping rate was >90%. Transcript assembly and quantification were performed with StringTie (v2.2.1), and gene-level read counts were obtained using HTSeq (v0.13.5). Expression levels were normalized as FPKM (Fragments Per Kilobase of transcript per Million mapped reads). Differential expression analysis was conducted using edgeR (v3.36.0). Genes with |log_2_(fold change)| ≥ 1 and adjusted *p*-value (padj) ≤ 0.05 (Benjamini–Hochberg correction) were considered significantly differentially expressed. GO and KEGG enrichment analyses were performed using clusterProfiler (v4.2.0).

The sequencing work was completed by Kaitai Biological Co., Ltd (Hangzhou, China). Using the statistics of sequencing error rate, data volume and comparison rate, the quality of sequencing was evaluated. Subsequently, the sequencing data were compared and screened to obtain the results of transcript quantitative and differential analysis, GO functional enrichment and KEGG pathway analysis.

### 2.14. Quantitative Real-Time PCR (qRT-PCR)

Total cellular RNA was extracted using a commercial RNA extraction kit, and reverse transcription was performed following the manufacturer’s protocol. The cDNA synthesis reaction conditions were 50 °C for 15 min, 85 °C for 5 min, followed by a hold at 4 °C. The resulting cDNA was appropriately diluted and subjected to SYBR Green-based quantitative PCR. The thermal cycling parameters were as follows: initial denaturation at 95 °C for 3 min, followed by 40 cycles of amplification at 95 °C for 5 s and annealing/extension at 60 °C for 30 s, and concluding with a melting curve analysis. Data were exported and analyzed using the comparative Ct (2 − ΔΔCt) method.

### 2.15. ROS Production Detection

The ROS level in C2C12 myotubes was measured using a DCFH-DA fluorescent probe with a Reactive Oxygen Species Assay Kit (S0033S, Beyotime, Shanghai, China). Briefly, DCFH-DA was diluted to 10 μM in serum-free culture medium. Cells were incubated with this working solution at 37 °C for 20 min in the dark, then washed three times with serum-free medium to remove excess probe. Then, cells were treated with Rosup (diluted 1:1000 in serum-free medium) for 20–30 min. Fluorescence was then detected using an excitation wavelength of 488 nm and an emission wavelength of 525 nm (FITC channel) by fluorescence microscopy.

### 2.16. Bioinformatics Prediction of Carnosine-Binding Targets

(1) Structural preparation: The two-dimensional (2D) molecular structure of carnosine was retrieved from the PubChem database and saved in SDF format. The three-dimensional (3D) crystal structures of phosphatidylinositol 3-kinase (PI3K) from both Mus musculus and Homo sapiens were downloaded from the RCSB Protein Data Bank (PDB).
(2) Molecular docking simulation: The 2D structure of carnosine and the 3D structures of target proteins (PI3K) were uploaded to the CB-DOCK2 online docking server. Molecular docking calculations were performed using the platform’s default parameters.
(3) Docking analysis: Following completion of docking simulations, the optimal binding conformations of carnosine with each target protein were extracted and visualized. Vina scores (binding energy values) were recorded to evaluate the binding affinity between carnosine and the respective targets.

### 2.17. Statistical Analysis

All experiments were independently repeated at least three times (biological replicates). Quantitative data from RT-qPCR and Western blotting analyses were expressed as mean ± standard deviation (SD) and were analyzed using GraphPad Prism version 9.5.0 (GraphPad Software, San Diego, CA, USA). For comparisons between two groups, an unpaired two-tailed Student’s *t*-test was used after confirming normality (Shapiro–Wilk) and equal variance (Levene’s test). For multiple groups, one-way or two-way ANOVA was performed, followed by Tukey’s post hoc test for all pairwise comparisons or Dunnett’s post hoc test for comparisons against a single control group.

Specifically, for RT-qPCR, six technical replicates were run per sample. For Western blot densitometry, bands were quantified using ImageJ, and ratios of target protein to loading control were calculated. For immunofluorescence quantification, five random fields per well were captured in a blinded manner (*n* = 3 wells per group), and average fluorescence intensity, myotube fusion index, and myotube diameter were calculated using the ImageJ Cell Counter plugin. The significance level was set to *p* < 0.05, * *p* < 0.05, ** *p* < 0.01, *** *p* < 0.001, and **** *p* < 0.0001 for the difference between the labeled samples.

## 3. Results

### 3.1. Establishment of STZ-Induced T2DM Sarcopenia Mouse Model

In order to avoid the interference of hormones, this study used male ICR mice to establish a T2DM sarcopenia mouse model. Firstly, 4-week-old mice were fed a high-fat diet for 1 month, followed by intraperitoneal injection of STZ for 5 consecutive days to induce T2DM. Before STZ administration, all groups exhibited normal blood glucose levels (approximately 5 mM). After STZ injection, blood glucose reached ≥11.1 mmol/L approximately 10 days after the last STZ injection, which confirmed T2DM.

**Figure 1:**
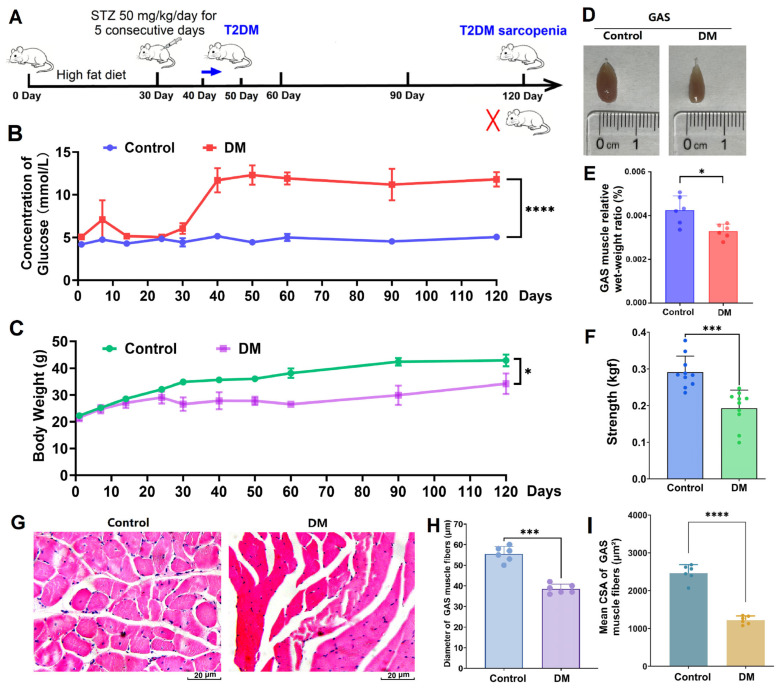
**Establishment of a STZ-induced T2DM sarcopenia mouse model.** (**A**) Methodological process for animal models. (**B**) Changes in blood glucose concentration in mice. (**C**) Changes in body weight in mice. Statistical analyses were performed using two-way repeated-measures analysis of variance (two-way RM ANOVA) with Geisser–Greenhouse correction for sphericity. (**D**) Images of the change in gastrocnemius muscle mass. (**E**) Change in gastrocnemius muscle wet weight ratio. (**F**) Changes in skeletal muscle strength. Forelimb pull strength was normalized to body weight, and data from 10 pulls per mouse were analyzed. (**G**) HE staining for gastrocnemius muscle. (**H**) Statistical data of the diameter of gastrocnemius muscle fibers. (**I**) Statistical data of gastrocnemius muscle fiber cross-sectional area (CSA). All animal experiments were performed with six mice per group, *n* = 6, **** *p* < 0.0001, *** *p* < 0.001, * *p* < 0.05.

Then, the mice were maintained for an additional 3 months to allow for the development of sarcopenia. During this 3-month maintenance period, blood glucose was always maintained at ≥11.1 mmol/L (Figure 1A,B). At the same time, the body weight results showed that the control mice grew normally, and the body weight continued to increase from about 20 g to about 40 g. Although the body weight of the mice in the experimental group increased in the early stage of the T2DM mouse model (the first 25 days of the experiment), the body weight was only maintained below 30 g (Figure 1C).

Subsequently, the gastrocnemius muscles of the mice were collected, weighed, and subjected to HE staining to evaluate skeletal muscle atrophy. The results showed that the muscle mass and wet weight ratio of the gastrocnemius muscles were significantly lower than those of the corresponding control group (Figure 1D–F). Meanwhile, HE staining results showed that compared with the control group, the muscle fiber diameter of the gastrocnemius muscle in the T2DM mice was significantly reduced, indicating that the continuous T2DM state led to skeletal muscle atrophy in the mice (Figure 1G,H). In summary, a T2DM sarcopenia mouse model was established. This model was then used for subsequent research.

### 3.2. Metabolomic Sequencing of T2DM Sarcopenia Mouse Model

**Figure 2:**
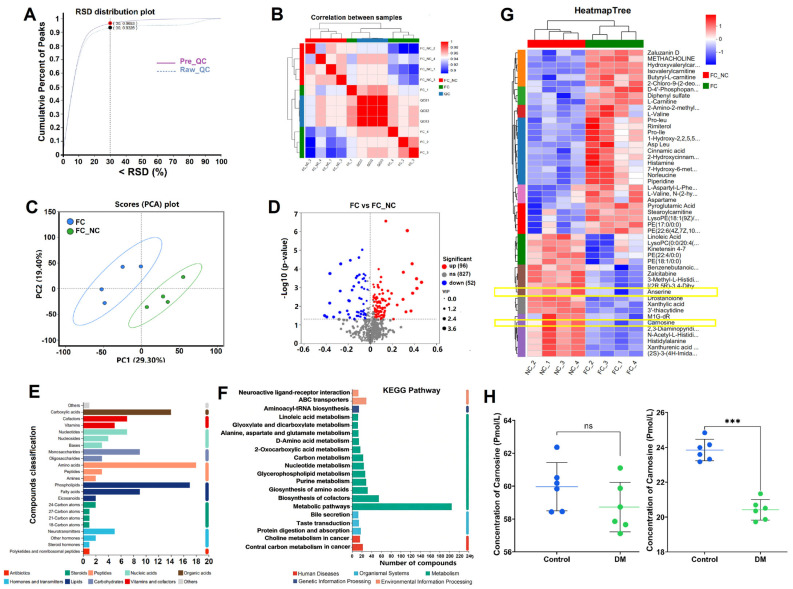
**Results and verification of metabolomic sequencing for skeletal muscle atrophy in STZ-induced T2DM mice.** (**A**) Sample quality control evaluation chart: abscissa = RSD value; ordinate = cumulative proportion of ion peaks. Blue dotted line denotes original data; purple solid line denotes preprocessed data. (**B**) Sample correlation heat map. Each grid represents a correlation between two samples, with color indicating relative magnitude of correlation coefficient. Clustering branch length reflects inter-sample relative distances; same-branch samples are highly similar. (**C**) After dimensionality reduction analysis, gastrocnemius muscle samples were mapped as points on principal component axes P1 and P2. Inter-point distances reflect sample similarity—closer points mean higher similarity, greater distances indicate larger differences. The 95% confidence ellipses delineate each group’s distribution ranges, with no outliers detected. (**D**) Volcano diagram of differential metabolites. Differential expression was determined based on the following criteria: |log_2_(fold change)| ≥ 1 and *p* < 0.05. (**E**) Statistical chart of KEGG differential metabolite compound classification. (**F**) Statistical chart of KEGG analysis of differential metabolites. (**G**) Results of heat map analysis of significantly different metabolites. The yellow box in the figure indicates the contents of carnosine and anserine in the gastrocnemius muscles of the experimental group were significantly decreased compared with those in the control group. (**H**) ELISA results of carnosine content in plasma and gastrocnemius muscle. FC_NC represents the control group, and FC represents the experimental group of T2DM sarcopenia. For metabolomics analysis (**A**–**G**), *n* = 4 per group and for ELISA (**H**), *n* = 6 per group. ns indicates no significant difference between groups, *** *p* < 0.001.

In order to explore the pathogenesis and prevention strategies of T2DM sarcopenia, metabolomics sequencing was performed on the gastrocnemius muscles of the control group and the experimental group obtained above (4 samples in each group). The results of metabolome sequencing quality control evaluation of the gastrocnemius muscles showed that over 96% of preprocessed metabolite peaks had RSD < 30%, indicating that most metabolites possessed stable signals and low impurity interference, and the overall data quality was qualified (Figure 2A). Sample correlation analysis showed that there was a good correlation between different samples within the control group and the experimental group (Figure 2B). The above results show that the gastrocnemius muscle extracts are of good quality and can be used for metabolomics sequencing research.

After non-targeted LC-MS metabolomics detection, the contents of metabolites in the gastrocnemius muscles of the control and experimental groups were obtained, as shown in Table S3. At the same time, the results of the CA (Correspondence Analysis) score plot showed that the similarity between different samples in the control group and the experimental group was high, and no abnormal samples appeared. Therefore, the annotated metabolites can be analyzed for differential content (Figure 2C).

Results of differential metabolite analysis: The composition of differential metabolites obtained by metabolome sequencing was analyzed. The results showed that compared with the control group, there was no significant change in the content of 827 metabolites, 96 metabolites were upregulated and 52 metabolites were downregulated in the experimental group (Figure 2D). The classification and distribution of all significant differential metabolites and the corresponding KEGG pathway annotation analysis showed that the classification of differential metabolites mainly focused on the changes in lipids, amino acids, and carbohydrates (Figure 2E). Enrichment analysis of the KEGG pathways involved in these differential metabolites showed that the most significant pathway was the metabolic pathway, and 220 differential metabolites were involved in the pathway changes (Figure 2F). There were significant metabolic changes in the gastrocnemius muscles of the T2DM sarcopenia mouse model.

The cluster analysis of specific differential metabolites showed that the contents of carnosine and anserine in the gastrocnemius muscles of the experimental group were significantly lower than those in the control group (Figure 2G). Both of them were imidazole dipeptides. Anserine, as a carnosine-methylated derivative, mainly exists in poultry and fish tissues, and the concentration in the human body is low. The distribution of carnosine is wider and the content is higher, which is very important for skeletal muscle function. It is speculated that the persistence of T2DM symptoms may lead to a decrease in carnosine content and cause skeletal muscle mass attenuation and dysfunction. Subsequently, the content of carnosine in the serum and gastrocnemius muscle of the mice was determined by ELISA. The results showed that, consistent with the results of metabolome sequencing, the content of carnosine in the gastrocnemius muscle of the mice decreased significantly. However, compared with the control group, there was no significant change in the content of carnosine in the serum of the experimental group (Figure 2H).

### 3.3. High-Glucose Affects the Expression of Carnosine Metabolism-Related Enzyme Genes in C2C12 Myotubes

**Figure 3:**
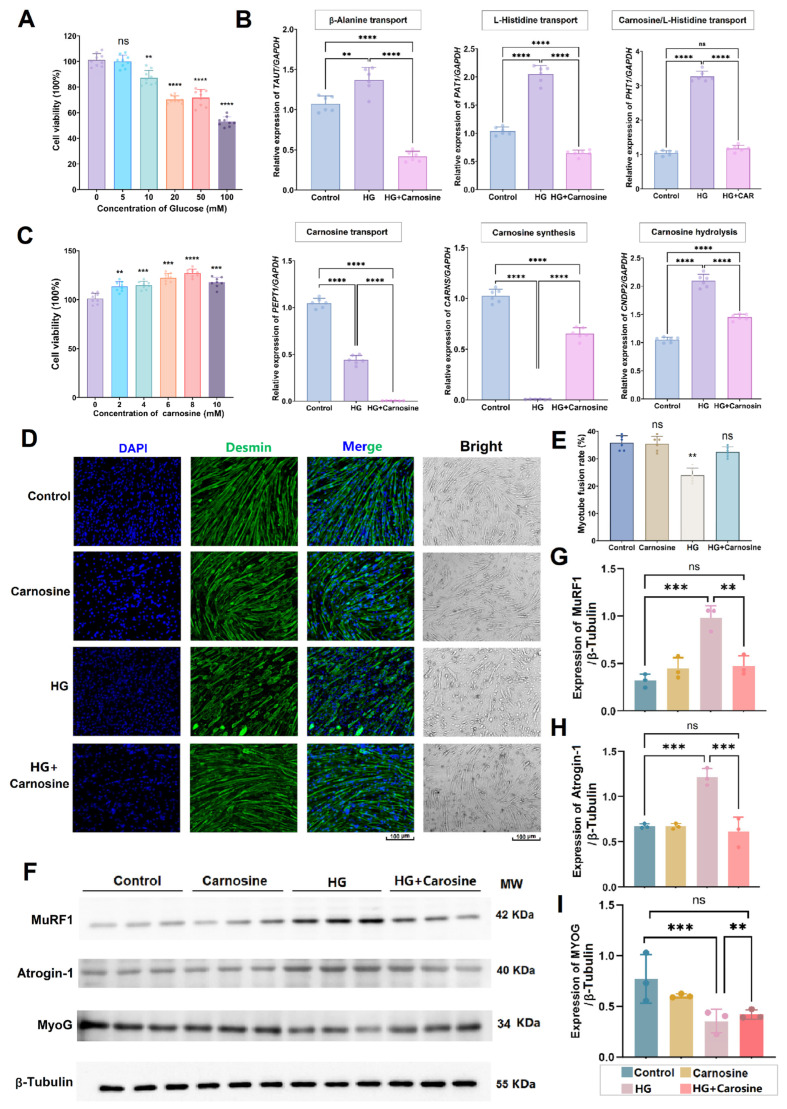
**Carnosine attenuates high-glucose-induced atrophy of C2C12 myotubes.** (**A**) C2C12 cell viability treated with glucose detected by CCK8 assay. (**B**) Expression of carnosine metabolism-related genes in C2C12 myotubes measured by SYBR Green quantitative RT-PCR. (**C**) C2C12 cell viability treated with carnosine detected by CCK8 assay. (**D**) Desmin immunofluorescence staining of myotubes treated with glucose. Green fluorescent signals represent Desmin, while blue fluorescent signals represent cell nuclei stained with DAPI. HG represents the treatment with 10 mM glucose. (**E**) Statistical results of myotube fusion rate. The statistical significance of each bar in (**D**) was compared with the corresponding 0 mM group. *n* = 6, ns indicates no significant difference, ** *p* < 0.01. (**F**) Western blotting images of MuRF1, Atrogin-1 and MyoG. (**G**–**I**) Statistical results of WB blot gray scanning. *n* = 3, ns indicates no significant difference between groups, *** *p* < 0.001, ** *p* < 0.01. In CCK8 assay results (**A**) and (**C**), the statistical significance of each bar was compared with the corresponding 0 mM group, *n* = 6, ns indicates no significant difference between groups, **** *p* < 0.0001, *** *p* < 0.001, ** *p* < 0.01. In SYBR Green quantitative RT-PCR results, *n* = 6, ns indicates no significant difference between groups, **** *p* < 0.0001, ** *p* < 0.01. HG denotes treatment with 10 mM glucose.

In order to explore the molecular mechanism of carnosine alleviating skeletal muscle atrophy in T2DM sarcopenic mice, a high-glucose-induced myotube atrophy model was established in this study—that is, different concentrations of glucose (5 mM, 10 mM, 20 mM, 50 mM, 100 mM) were added to the C2C12 myotube culture system to induce myotube atrophy. The results of CCK-8 assay showed that high concentration of glucose could affect the viability of C2C12 cells. When the concentration of glucose reached 100 mM, the cell viability even decreased to about 50% (Figure 3A). Therefore, this study used 10 mM glucose to act on C2C12 myotubes to simulate skeletal muscle atrophy caused by high-glucose.

The metabolic homeostasis of carnosine in skeletal muscle tissue is affected by the expression of a variety of related metabolic genes: *TauT* on the skeletal muscle cell membrane is responsible for the transport of β-alanine, *PAT1* is responsible for the transport of L-histidine, *PHT1* is responsible for non-specific transport of carnosine or L-histidine, and *PEPT1* is responsible for specific transport of carnosine. When the raw materials β-alanine and L-histidine are transported to the cells, the two are catalyzed by the carnosine synthase CARNS to synthesize carnosine, and the CNDP2 in the cytoplasm will decompose carnosine to maintain the stability of carnosine content in skeletal muscle cells. In view of the complexity of metabolism in vivo, this study used a 10 mM glucose-treated C2C12 myotube model to detect the expression of carnosine-related metabolic enzyme genes, aiming to explore the effect of high-glucose on the metabolic homeostasis of carnosine. The results showed that (1) after high-glucose treatment, the expression of *TauT*, *PAT1* and *PHT1* genes was significantly upregulated compared with the corresponding control group, suggesting that it may be the “emergency uptake” of amino acids by myotubes under high-glucose; (2) the expression of carnosine-specific transporter gene *PEPT1* in the high-glucose group was significantly lower than that in the control group, and the low level was not restored after the supplementation of carnosine, suggesting that high-glucose could directly inhibit the specific uptake of carnosine by myotubes; and (3) after high-glucose treatment, the expression of the carnosine synthase *CARNS* gene was significantly downregulated, and the expression of *CNDP2* gene was significantly upregulated, suggesting that high-glucose inhibits carnosine synthesis and enhances carnosine decomposition, which may lead to a decrease in intracellular carnosine content (Figure 3B). In summary, high-glucose may disturb the uptake of β-alanine, L-histidine and carnosine in skeletal muscle cells; inhibit the synthesis of carnosine; promote its decomposition; and lead to the imbalance of carnosine metabolic homeostasis. The mechanism and inducement need to be further explored and a maintenance strategy developed for targeting carnosine metabolic homeostasis.

In order to clarify whether carnosine can alleviate C2C12 myotube atrophy caused by high-glucose, different concentrations of carnosine (2 mM, 4 mM, 6 mM, 8 mM, 10 mM) were applied to C2C12 cells in this study. The results of CCK8 showed that 8 mM carnosine could significantly upregulate the viability of C2C12 cells (Figure 3C). In this study, 8 mM carnosine was added to the C2C12 myotube culture system, and the myotube fusion rate was identified by immunofluorescence staining of Desmin protein. Statistical results showed that 10 mM glucose significantly reduced the fusion rate of C2C12 myotubes, while carnosine treatment could alleviate high-glucose-induced myotube atrophy (Figure 3D,E). At the same time, Western blotting results showed that the protein expression levels of skeletal muscle atrophy marker molecules MuRF1 and Atrogin-1 in C2C12 myotubes after 10 mM high-glucose treatment were significantly higher than those in the control group. After carnosine treatment, the protein expression levels of MuRF1 and Atrogin-1 were downregulated compared with the high-glucose treatment group. Moreover, when treated with high-glucose, the protein expression of MyoG, a marker molecule of skeletal muscle maturation and differentiation, decreased significantly, while the expression level of MyoG was significantly upregulated after supplementation with carnosine (high-glucose + carnosine) compared with the high-glucose treatment group (Figure 3F–I). In summary, the addition of carnosine can alleviate C2C12 myotube atrophy.

### 3.4. Transcriptome Sequencing of Carnosine Alleviating C2C12 Myotube Atrophy in Mice

In order to further explore the molecular mechanism of carnosine alleviating the atrophy of C2C12 myotubes in mice, high-throughput transcriptome sequencing was performed on myotubes treated with high-glucose and high-glucose combined with carnosine (3 sample replicates in each group). Among them, the saturation of sequencing represents the relationship between the amount of sequencing and the number of detected genes. As the amount of sequencing increases, the number of detected genes tends to a stable value. The saturation curve analysis of the gene expression in myotube samples showed that the curves of different samples tended to be gentle, indicating that the sequencing depth could cover most of the known splicing connections, and the sequencing saturation between samples was stable and could be used for subsequent analysis (Figure S1). Subsequently, the raw data were filtered, the sequencing error rate was checked, and the GC content distribution was checked to obtain the clean reads used for subsequent analysis. The transcription sequencing data are summarized in Table S4.

**Figure 4:**
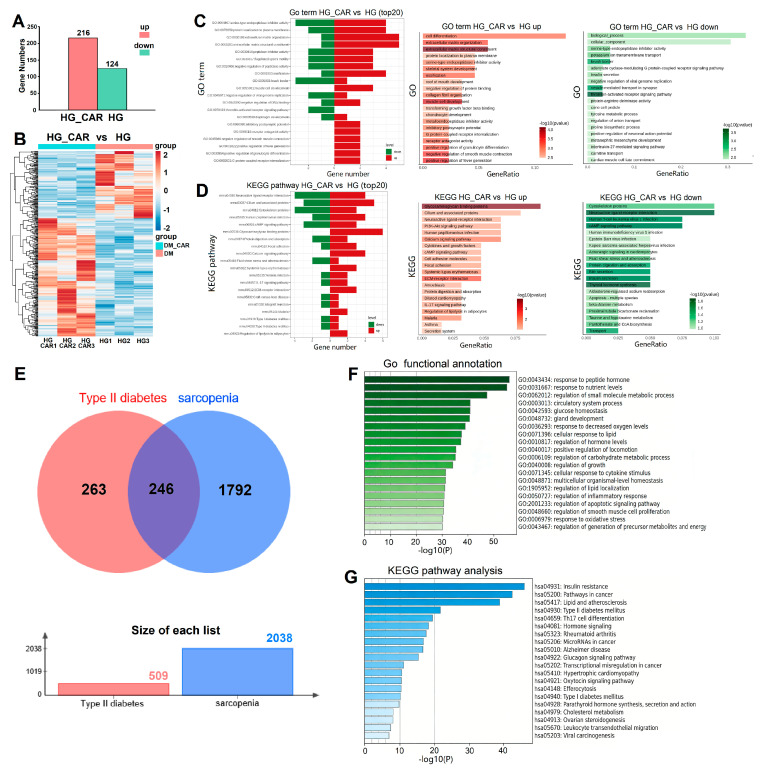
**Results of transcriptome sequencing on carnosine attenuating atrophy of C2C12 myotubes and bioinformatics prediction of common disease targets for T2DM and sarcopenia.** (**A**) Statistical bar chart of the number of differentially expressed genes. (**B**) Heatmap: X-axis = samples; Y-axis = differential genes. Genes are clustered by expression similarity, with expression levels increasing from blue to red. (**C**) Results of GO term enrichment analysis for significant differentially expressed genes. (**D**) Results of KEGG analysis on significantly differentially expressed genes. HG refers to myotubes treated with 10 mM glucose, while HG_CAR refers to myotubes treated with 10 mM glucose combined with 8 mM carnosine treatment. (**E**) Results of bioinformatics prediction for common disease targets of T2DM and sarcopenia. Venn diagram of statistics for common disease targets of T2DM and sarcopenia. (**F**) Results of GO term analysis of common disease targets of T2DM and sarcopenia. (**G**) Results of KEGG analysis of common disease targets of T2DM sarcopenia. For transcriptome sequencing, *n* = 3 biological replicates per group.

In this study, edgeR software was used to analyze the significance of expression differences, and genes with a pvalue and padj of less than 0.05 and a |log_2_FoldChange| of greater than 2 were considered significantly differentially expressed. The number of differential genes in each group was counted. The results showed that compared with the high-glucose treatment group, 216 genes were significantly upregulated and 124 genes were significantly downregulated in the myotubes of the high-glucose + carnosine treatment group (Figure 4A). In addition, the log_10_ (FPKM + 1) value was converted (scale number) and cluster analysis was performed. At the same time, the R language ggbio package (1.61.0) was used to label RNA on the genome according to the genomic information, the overall expression of RNA, and the results of differential RNA expression analysis, and the differential gene hierarchical clustering heat map was obtained (Figure 4B).

SYBR Green quantitative RT-PCR was used to verify that the expression patterns of differentially expressed genes matched the transcriptome sequencing results. Several genes involved in skeletal muscle growth and metabolism, including *Pik3c2g*, *Perm1*, *Atp1a4*, and *Wfikkn1*, were selected. Their expression levels were all significantly increased in the high-glucose + carnosine treatment groups compared with the high-glucose control groups (Figure S2). These results confirmed the accuracy of the transcriptome sequencing results.

In this study, GO functional enrichment analysis was performed on significantly differentially expressed genes. The results showed that compared with the high-glucose treatment group, biological processes such as cell differentiation, skeletal muscle development, and extracellular matrix organization in the myotubes of the high-glucose + carnosine treatment group were significantly changed, suggesting that carnosine treatment can affect skeletal muscle cell function (Figure 4C). KEGG analysis of significantly differentially expressed genes showed that after carnosine treatment, the upregulated differentially expressed genes were mainly enriched in the PI3K signaling pathway, which was one of the core pathways of cell physiology (Figure 4D). Combined with the results of GO functional enrichment analysis, it was speculated that carnosine may alleviate high-glucose-induced C2C12 myotube atrophy by activating the PI3K signaling pathway.

In addition, this study collected related targets of human diabetes and sarcopenia from three authoritative human disease target databases (CTD, GeneCards and OMIM). After screening, common targets of the two diseases were predicted by Venn diagram analysis. The results showed that a total of 246 common target genes were obtained (Figure 4E). After GO functional enrichment analysis and KEGG pathway analysis of these common disease targets, it was found that the KEGG pathways involved in the above genes were mainly insulin resistance, glucose metabolism homeostasis, and T2DM pathways. Among them, PI3K signaling pathway is the core regulator of these pathways (Figure 4F,G).

### 3.5. Carnosine Promotes Mitochondrial Biosynthesis in C2C12 Myotubes Under High-Glucose Treatment

**Figure 5:**
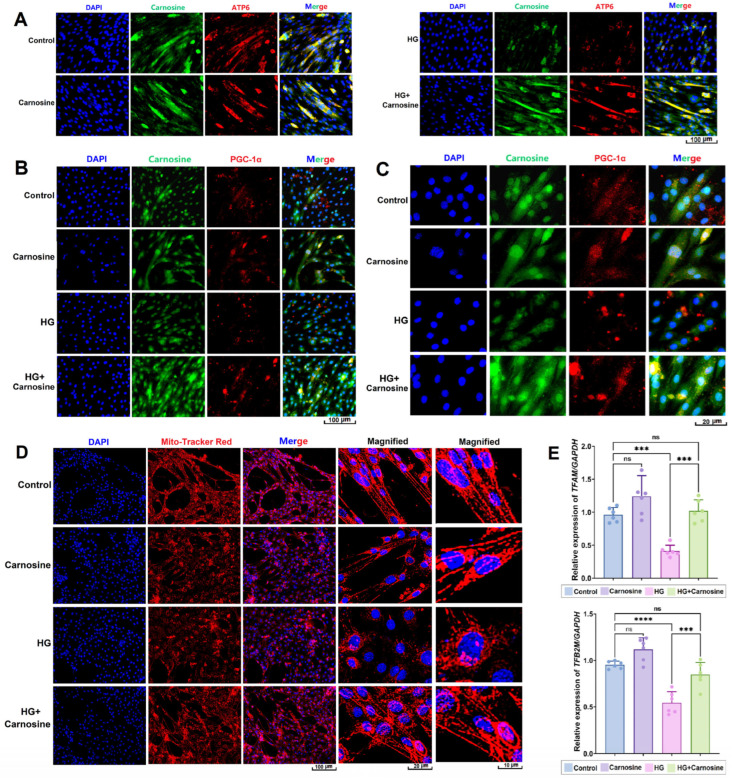
**Carnosine promotes mitochondrial biogenesis in C2C12 myotubes under high-glucose treatment.** (**A**) Immunofluorescence staining results for carnosine and ATP6. Blue signals represent DAPI-stained nuclei, green signals indicate carnosine, and red signals indicate ATP6. (**B**,**C**) Immunofluorescence staining results for carnosine and PGC-1α. Blue signals represent DAPI-stained nuclei, green signals indicate carnosine, and red signals indicate PGC-1α. (**D**) Mitochondrial staining results of myotubes using Mito-Tracker Red probe. Blue signals represent DAPI-stained nuclei, and red signals indicate mitochondria. (**E**) SYBR Green quantitative RT-PCR results for *TFAM* and *TFB2M* genes related to mtDNA transcription. HG denotes treatment with 10 mM glucose in this figure. For immunofluorescence staining (**A**–**D**), *n* = 3 biological replicates; for qRT-PCR (**E**), *n* = 6 biological replicates. ns indicates no significant difference between groups, **** *p* < 0.0001, *** *p* < 0.001.

The results of the transcriptome sequencing of C2C12 myotubes showed that under high-glucose conditions, the transcription level of the mitochondrial *ATP6* gene was significantly upregulated after adding carnosine compared with the control group (the fold change was 2.9), suggesting that carnosine may affect mitochondrial function. Therefore, this study first used immunofluorescence staining to clarify the subcellular localization of carnosine and ATP6 (mitochondrial respiratory chain complex V subunit) in C2C12 myotubes. ATP6 protein is a key component of the electron respiratory chain on the mitochondrial inner membrane. The staining results showed that the ATP6 fluorescence signal (red) in the myotubes was significantly reduced under high-glucose treatment, suggesting that high-glucose may damage the myotube mitochondria; after the addition of carnosine, the positive signal of ATP6 in the myotubes was significantly increased compared with the high-glucose group, and recovered to a level roughly consistent with the control group or the carnosine group (Figure 5A), indicating that carnosine may play a role in regulating the number and function of mitochondria in myotubes. In addition, after high-glucose treatment, the green fluorescence signal intensity and distribution range of carnosine in myotubes decreased (Figure 5A), suggesting that high-glucose may destroy the metabolic homeostasis of carnosine in skeletal muscle cells.

The above results suggest that carnosine may play a role in regulating the number of mitochondria and their function. Therefore, this study further explored the effect of carnosine on the subcellular localization and distribution of PGC-1α. PGC-1α is a core transcriptional coactivator that regulates mitochondrial biosynthesis, energy metabolism, and skeletal muscle function. It is also a key node molecule in the PI3K/AMPK/PGC-1α signaling pathway. The results of immunofluorescence staining showed that PGC-1α (red fluorescence signal) could be transferred from the cytoplasm to the nucleus in myotubes treated with carnosine under high-glucose conditions, suggesting that carnosine could promote the nuclear translocation of PGC-1α and promote mitochondrial biosynthesis (Figure 5B,C). In addition, it is worth noting that carnosine (green fluorescence signal) is distributed in the nucleus in addition to the cytoplasm; after exogenous addition of carnosine, the fluorescence signal of carnosine in the nucleus was significantly enhanced (Figure 5B,C). However, the relationship between carnosine in the nucleus and PGC-1α nuclear translocation, and the specific mechanism of regulating the number and function of mitochondria need to be further studied.

In order to further verify the regulatory effect of carnosine on high-glucose-induced mitochondrial biosynthesis in myotubes, this study used the mitochondrial-specific live cell probe Mito-Tracker Red to observe the mitochondrial distribution (red fluorescence signal) in myotubes. The results showed that after high-glucose treatment, the intensity of the Mito-Tracker Red fluorescence signal in the myotubes decreased and the distribution range narrowed, suggesting that the number of mitochondria may decrease. However, high-glucose + carnosine treatment significantly reversed the above changes, increased the intensity of mitochondrial fluorescence signal and expanded the distribution range, suggesting that carnosine could counteract the decrease in mitochondrial number induced by high-glucose (Figure 5D). It is worth noting that the mitochondria in the myotubes of the high-glucose treatment group are mainly in a split state, while the mitochondria in the high-glucose + carnosine treatment group are mainly in a fusion state; mitochondrial fusion can promote mitochondrial function integration and biosynthesis, suggesting that carnosine may also regulate mitochondrial dynamic balance. Whether carnosine can also help maintain the integrity of mitochondrial inner membrane cristae is also worthy of further exploration.

In order to clarify whether carnosine can further promote mitochondrial biosynthesis while promoting nuclear translocation of PGC-1α, the mRNA expression levels of key mitochondrial DNA transcription factors *TFAM* and *TFB2M* were detected by fluorescence quantitative RT-PCR. The results showed that compared with the control group, the expression levels of *TFAM* and *TFB2M* genes in myotubes treated with high-glucose were significantly decreased. Compared with the high-glucose treatment group, the expression of *TFAM* in the high-glucose + carnosine treatment group was significantly upregulated, but there was no significant difference in the expression of *TFB2M* (Figure 5E). In summary, carnosine may upregulate *TFAM* gene transcription by regulating the activity of PGC-1α in C2C12 myotubes, thereby promoting mitochondrial biosynthesis.

Additional functional assays related to mitochondrial function were conducted in this study. mtDNA copy number (*mt-Atp6*, ATP synthase F0 subunit 6; *mt-Cytb*, Cytochrome b; *mt-Nd1*, NADH dehydrogenase subunit 1; *mt-Nd4*, NADH dehydrogenase subunit 4) and ATP content in C2C12 myotubes following carnosine treatment under high-glucose were both significantly increased compared with the high-glucose treatment control group (Figures S3 and S4). Meanwhile, the carnosine treatment decreased ROS production (Figure S5). These data indicate that carnosine could promote mitochondrial biogenesis, optimize bioenergetic function, and alleviate oxidative stress.

### 3.6. Carnosine Alleviates C2C12 Myotube Atrophy Through the PI3K/AMPK/PGC-1α Signaling Pathway

**Figure 6:**
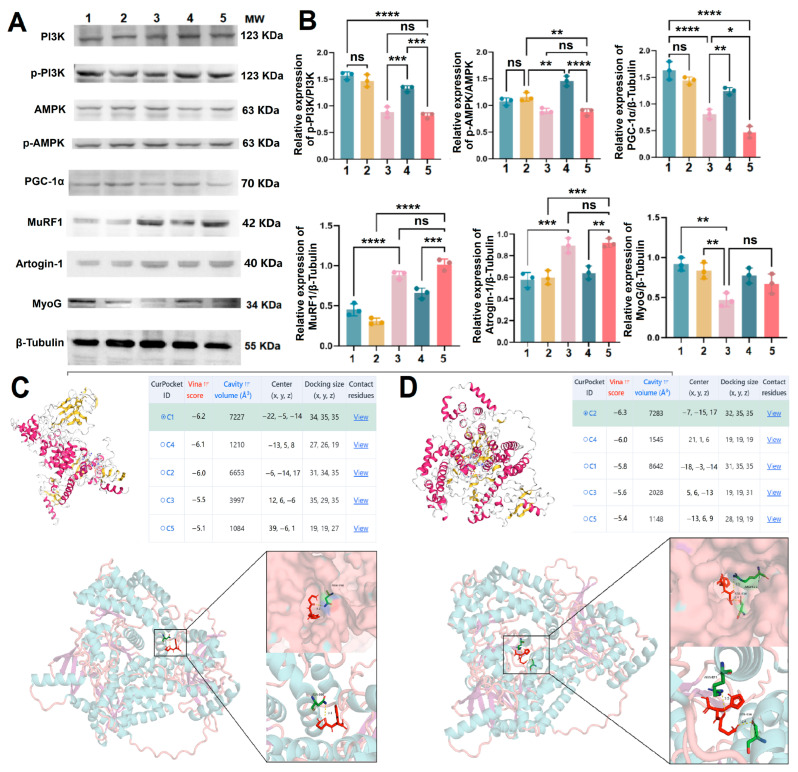
**Western blotting for carnosine alleviating C2C12 myotube atrophy via the PI3K/AMPK/PGC-1α signaling pathway.** (**A**) Western blotting images of PI3K/AMPK/PGC-1α signal pathway, MuRF1, Atrogin-1, and MyoG. (**B**) Statistical results of WB blot gray scanning. *n* = 3, ns indicates no significant difference between groups, **** *p* < 0.0001, *** *p* < 0.001, ** *p* < 0.01, * *p* < 0.05. (**C**) Bioinformatics prediction of molecular docking for carnosine binding to PI3K in mice. (**D**) Bioinformatics prediction of molecular docking for carnosine binding to PI3K in humans.

Based on the above transcriptome sequencing and bioinformatics prediction results of the disease targets, this study used PI3K inhibitor LY294002 to block the PI3K pathway to explore the relationship between carnosine and PI3K signaling and C2C12 myotube atrophy. Western blotting results showed that when C2C12 myotubes were treated with high-glucose, the protein expression levels of p-PI3K, p-AMPK, PGC-1α and MyoG were significantly decreased compared with the corresponding control group, indicating that high-glucose treatment downregulated the PI3K/AMPK/PGC-1α signaling pathway and led to myotube atrophy. When carnosine was supplemented, the protein expression levels of p-PI3K, p-AMPK, PGC-1α and MyoG were significantly upregulated compared with the corresponding high-glucose treatment group, indicating that carnosine could activate the PI3K/AMPK/PGC-1α signaling pathway and resist myotube atrophy. However, after treatment with PI3K inhibitor LY294002, the activity of carnosine activating the PI3K/AMPK/PGC-1α signaling pathway and the effect of resistance to myotube atrophy were reduced (Figure 6A,B). The above results indicate that carnosine alleviates C2C12 myotube atrophy through the PI3K/AMPK/PGC-1α signaling pathway. The above results show that after blocking PI3K, carnosine can no longer activate its downstream AMPK phosphorylation and PGC-1α expression, indicating that PI3K may be a target molecule for carnosine to alleviate high-glucose-induced skeletal muscle atrophy.

In addition, this study also predicted the binding of carnosine to mouse and human PI3K proteins by bioinformatics methods such as molecular docking and small molecule–protein binding energy calculation. The results showed that carnosine could bind to mouse (p110β subunit, UniProt number: Q8BTI9, PK3CB_MOUSE) and human (p110β subunit, UniProt number: P42338, PK3CB_HUMAN) PI3K key catalytic subunit p110β (Figure 6C,D). The binding energy range was about −5 kcal/mol to −6 kcal/mol, indicating that carnosine has a good binding potential with PI3K protein. However, it is still necessary to verify the actual binding of carnosine to PI3K by means of biological interference molecular interaction instrument so as to truly clarify the disease target of carnosine in the treatment of T2DM sarcopenia and provide theoretical support for its application strategy.

### 3.7. Carnosine Alleviates Skeletal Muscle Atrophy in T2DM Sarcopenic Mice

**Figure 7:**
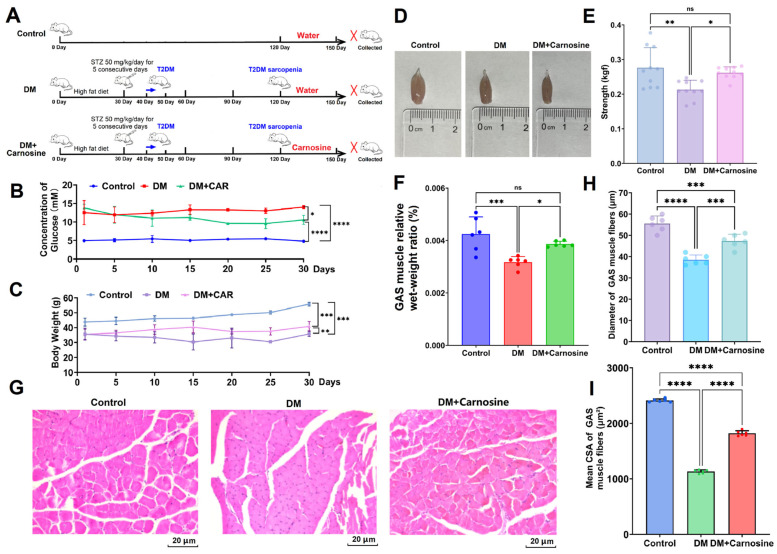
**Effects of carnosine on blood glucose and body weight in a T2DM sarcopenia mouse model.** (**A**) Methodological process for animal models. (**B**) Changes in blood glucose concentration in mice. (**C**) Changes in body weight in mice. (**D**) Images of the change in gastrocnemius muscle mass. (**E**) Changes in skeletal muscle strength. (**F**) Change in gastrocnemius muscle wet weight ratio. (**G**) HE staining for gastrocnemius muscle. (**H**) Statistical data of the diameter of gastrocnemius muscle fibers. (**I**) Statistical data of gastrocnemius muscle fiber cross-sectional area (CSA). All animal experiments were performed with six mice per group, *n* = 6, ns indicates no significant difference between groups, **** *p* < 0.0001, *** *p* < 0.001, ** *p* < 0.01, * *p* < 0.05.

When T2DM occurs, the skeletal muscle tissue of mice undergoes atrophy, and the content of carnosine in the gastrocnemius muscle decreases significantly, suggesting that skeletal muscle atrophy in T2DM sarcopenia mice may be related to the decrease in carnosine content. In order to confirm this inference, this study implemented exogenous carnosine supplementation intervention in T2DM sarcopenia mice. Using a T2DM sarcopenia mouse model, the mice in the experimental group were given oral carnosine treatment for 30 consecutive days, and the blood glucose and body weight changes of the mice were recorded. The results of blood glucose monitoring showed that compared with T2DM mice, the blood glucose level of mice in the oral carnosine treatment group (DM + carnosine) decreased, and the body weight increased (Figure 7A–C). In summary, oral carnosine may play a role in alleviating the symptoms of T2DM. The weight of the gastrocnemius muscles and the tension of the mice were measured. The results showed that compared with T2DM mice, the muscle tension and gastrocnemius muscle weight of the T2DM carnosine treatment mice were significantly increased after carnosine treatment (Figure 7D–G). At the same time, the results of HE staining were consistent with this—that is, the muscle fibers of T2DM mice treated with carnosine were significantly higher than those of T2DM mice (Figure 7H,I). In summary, oral carnosine can alleviate skeletal muscle atrophy in T2DM sarcopenic mice.

**Figure 8:**
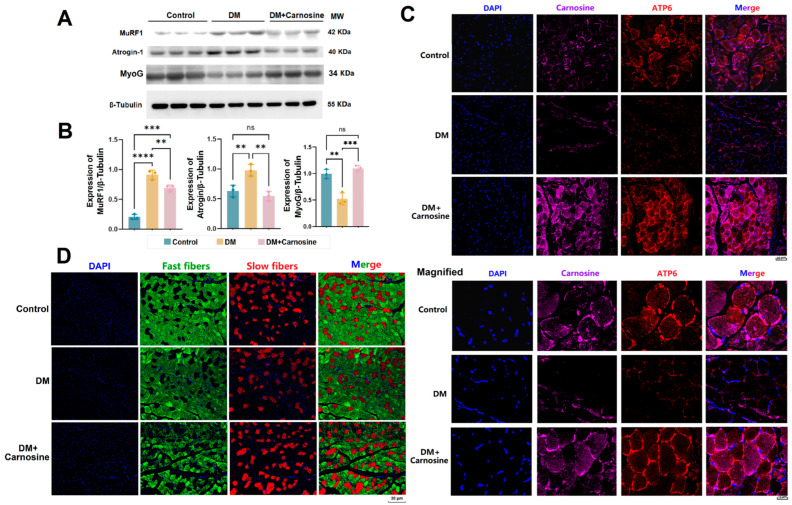
**Molecular mechanism of carnosine attenuating skeletal muscle atrophy in T2DM sarcopenia mice.** (**A**) Western blotting images of MuRF1, Atrogin-1 and MyoG. (**B**) Statistical results of WB blot gray scanning. *n* = 3, ns indicates no significant difference between groups, **** *p* < 0.0001, *** *p* < 0.001, ** *p* < 0.01. (**C**) Results of immunofluorescence staining for colocalization of carnosine and ATP6 in mice gastrocnemius muscle. The blue signals represent the nuclei stained with DAPI, the purple signals indicate carnosine, and the red signals indicate ATP6. (**D**) Results of immunofluorescence staining for myofiber types in mouse gastrocnemius muscle. The blue signals represent the nuclei stained with DAPI, the green signals indicate fast fibers, and the red signals indicate slow fibers. For immunofluorescence staining (**C**,**D**), *n* = 6 biological replicates.

In this study, Western blotting was used to detect the protein expression levels of skeletal muscle atrophy marker molecules Atrogin-1 and MuRF1. The results showed that compared with the control group, the expression levels of Atrogin-1 and MuRF1 proteins in T2DM mice were significantly upregulated, indicating that skeletal muscle underwent protein degradation and atrophy. The expression levels of Atrogin-1 and MuRF1 protein in T2DM sarcopenia mice treated with carnosine were significantly lower than those in T2DM mice. At the same time, when T2DM occurred, the protein expression of MyoG, a marker molecule of skeletal muscle maturation and differentiation, decreased significantly, while after the supplementation of carnosine, the expression level of MyoG was significantly upregulated compared with the T2DM group (Figure 8A,B). The above results indicate that exogenous supplementation of carnosine can alleviate skeletal muscle atrophy in T2DM sarcopenic mice. In order to further clarify whether carnosine can alleviate T2DM sarcopenia by promoting mitochondrial biosynthesis, this study used immunofluorescence staining to detect the expression and distribution of mitochondrial marker protein ATP6 in mouse gastrocnemius muscle. The results showed that ATP6 (red fluorescence signal) was widely distributed in the gastrocnemius muscle of the carnosine-treated group compared with the T2DM model mice (Figure 8C), suggesting that the addition of carnosine can increase the number of mitochondria in the gastrocnemius muscle. The results are consistent with the results of the in vitro myotube experiments shown in Figure 5A. In the pathological process of T2DM sarcopenia, skeletal muscle is often accompanied by mitochondrial dysfunction, which in turn induces the conversion of muscle fibers from slow-twitch oxidation type (high mitochondrial content and active oxidative metabolism) to fast-twitch glycolytic type (low mitochondrial content and glycolytic metabolism), and ultimately manifests as muscle mass loss and decreased contractile function. Based on this, by detecting the number of mitochondria and their distribution, this study further carried out double immunofluorescence staining identification of muscle fiber types in gastrocnemius muscle (mixed skeletal muscle, both fast and slow muscle fiber subtypes). The results showed that the gastrocnemius muscle of T2DM model mice was dominated by fast muscle fibers (green fluorescence signal). When treated with carnosine supplementation, the proportion of slow muscle fibers in the gastrocnemius muscle was significantly increased (Figure 8D). In summary, carnosine may alleviate muscle atrophy and dysfunction in T2DM sarcopenic mice by regulating the number of mitochondria and their function.

## 4. Discussion

In this study, STZ combined with high-glucose was used to construct a mouse T2DM model. Two consecutive fasting blood glucose (FBG) > 11.1 mmol/L were used as the standard for successful modeling. Skeletal muscle injury was induced by continuous maintenance of T2DM symptoms for 3 months. By detecting the wet weight ratio, diameter and pathological morphology of gastrocnemius muscle, skeletal muscle atrophy was confirmed, and the T2DM sarcopenia mouse model was successfully constructed (Figure 1). In order to explore the pathogenesis of T2DM sarcopenia and prevention strategies, this study then conducted a metabolomics study on the gastrocnemius muscle of T2DM sarcopenia mice. The results showed that the content of carnosine in the skeletal muscle of T2DM sarcopenia mice was significantly reduced, suggesting that it may be related to the occurrence of sarcopenia (Figure 2). In order to verify this hypothesis, this study conducted exogenous carnosine supplementation experiments in a T2DM sarcopenia mouse model and a high-glucose-induced C2C12 myotube atrophy model. The results of in vitro experiments showed that compared with the high-glucose treatment group, carnosine intervention could significantly improve C2C12 myotube atrophy and increase myotube fusion rate. At the same time, the protein expression of MuRF1 and Atrogin-1, the markers of skeletal muscle atrophy, was downregulated, and the expression of MyoG, the key molecule of muscle differentiation, was upregulated, indicating that carnosine could play an anti-muscle atrophy role by inhibiting skeletal muscle protein degradation and promoting muscle differentiation (Figure 3). Because C2C12 myotubes can simulate mature skeletal muscle cells and are not disturbed by complex environments in vivo, they are a classic model for studying skeletal muscle physiological functions. In this study, high-throughput transcriptome sequencing of C2C12 myotubes treated with high-glucose and carnosine intervention was further performed to explore its molecular mechanism.

Transcriptome sequencing results showed that carnosine intervention significantly activated the PI3K signaling pathway compared with the high-glucose group. As a core regulator of cell life activities, PI3K participates in various physiological processes, such as cell proliferation, differentiation, and apoptosis, through a complex signal network. Its abnormalities are closely related to diabetes, cancer, immune diseases, neurological diseases, and cardiovascular diseases [20]. This suggests that the PI3K signaling pathway may be a key mechanism for carnosine to alleviate sarcopenia in T2DM. According to the structural characteristics, substrate specificity and activation mode, PI3K can be divided into three categories, of which class I PI3K is the most closely related to physiological and pathological processes, and the research is the most in depth. Class I PI3K is further divided into subtypes IA and IB: IA (catalytic subunit p110α/β/δ, regulatory subunit p85) is activated by receptor tyrosine kinases (such as insulin receptors, growth factor receptors) [21], and the p110β subtype plays a central role in skeletal muscle metabolism regulation. Therefore, in this study, molecular docking was used to simulate the interaction of carnosine and mouse with human p110β protein. The results showed that carnosine had high binding energy with these two proteins (Figure 6C,D), suggesting that carnosine may activate the PI3K pathway by targeting p110β.

In order to further verify the in vivo intervention effect of carnosine, this study intervened in T2DM sarcopenia mice by oral administration of carnosine for 30 consecutive days. The results showed that the muscle tension and gastrocnemius muscle weight of T2DM sarcopenia mice were significantly increased after exogenous supplementation of carnosine. HE staining results also confirmed this effect. The muscle fiber diameter of carnosine-treated mice was significantly larger than that of untreated T2DM mice, indicating that carnosine can effectively alleviate T2DM-induced skeletal muscle atrophy. At the same time, it was found that carnosine intervention also improved the physiological and metabolic status of the mice, not only increasing body weight, but also significantly reducing the blood glucose level of T2DM mice (Figure 7).

Insulin resistance is the core pathological feature of T2DM, which refers to the decrease in insulin sensitivity of cells, which in turn leads to hyperglycemia and hyperinsulinemia [22]. When blood glucose continues to rise, insulin binds to the insulin receptor (IR) on the cell surface and induces autophosphorylation of the downstream insulin receptor substrate (IRS) [23]. As the first identified member of the IRS family, IRS-1 is widely expressed in muscle, fat and other tissues, and is a key molecule that mediates insulin and insulin-like growth factor-1 (IGF-1) signal transduction [24]. After phosphorylation of IRS-1, its specific tyrosine residues can bind to the PI3K regulatory subunit p85, thereby activating PI3K [25]. Activation of PI3K can trigger multiple downstream branch pathways to synergistically regulate skeletal muscle function: (1) PI3K/AKT/GLUT4 pathway: 90% of insulin-stimulated glucose utilization occurs in skeletal muscle. This pathway enhances glucose uptake and transport by promoting GLUT4 cell membrane translocation, while regulating glycogen synthesis and protein synthesis to maintain skeletal muscle metabolic homeostasis [26,27]; if the pathway is blocked, the GLUT4 translocation ability will decrease, which will aggravate insulin resistance [28]. (2) PI3K/AKT/mTOR pathway: Phosphorylation of ribosomal protein S6 kinase 1 (S6K1) and eukaryotic translation initiation factor 4E-binding protein 1 (4E-BP1) promotes skeletal muscle protein synthesis [29]. When the intracellular energy is insufficient, AMPK can activate the PI3K/AKT pathway by phosphorylating IRS-1 [30]. At the same time, it phosphorylates and stabilizes the key factor PGC-1α of mitochondrial biosynthesis, promotes the expression of mitochondrial transcription factors TFAM and TFB2M, and initiates mitochondrial biosynthesis. Mitochondria, as the “energy factory” of cells, can increase ATP synthesis efficiency, promote the conversion of fast muscle fibers to slow muscle fibers, and enhance skeletal muscle endurance and anti-fatigue ability. However, insufficient mitochondrial biosynthesis is an important reason for skeletal muscle mass loss and functional decline.

In order to clarify whether carnosine regulates mitochondrial biosynthesis through the PI3K signaling pathway, LY294002 was used to inhibit PI3K activity in a C2C12 myotube model in vitro. The results showed that LY294002 inhibitor reversed the alleviation effect of carnosine on high-glucose-induced myotube atrophy (Figure 6A,B), which proved that the anti-atrophy effect of carnosine was dependent on the PI3K pathway. It is suggested that PI3K pathway may be influenced by carnosine. Whether carnosine also improves T2DM sarcopenia by regulating the PI3K/AKT/GLUT4 and PI3K/AKT/mTOR signaling pathways to affect glucose uptake and protein metabolism in skeletal muscle cells remains to be further explored. This study did not assess these PI3K downstream effectors (e.g., AKT, mTOR, IRS-1, or GLUT4). Future investigations are therefore required to determine whether carnosine influences insulin signaling and glucose metabolism through these pathways.

Based on the results of transcriptome sequencing, carnosine could significantly up-regulate the expression of the *mt-ATP6* gene (Figure 4A,B). Therefore, in this study, live cell mitochondrial probe staining was performed on the myotubes, and ATP6 protein immunofluorescence staining was performed on the gastrocnemius muscle. The results showed that in both in vitro and in vivo experiments, carnosine intervention significantly increased the number of mitochondria, improved mitochondrial distribution, and up-regulated ATP6 protein expression (Figure 5A and Figure 8C). As the core subunit of mitochondrial electron respiratory chain complex V, ATP6 is directly involved in ATP synthesis in cell energy metabolism. Mutations in the *mt-ATP6* gene encoding this protein are associated with motor coordination dysfunction and developmental delay [31]. This type of phenotype is common in mitochondrial myopathy, encephalopathy and other diseases closely related to mitochondrial dysfunction. This suggests that carnosine may also have a potential role in improving mitochondrial myopathy and encephalopathy. In addition, in vitro mitochondrial probe staining showed that the myotube mitochondria in the high-glucose group were mainly divided, while the mitochondria showed a fusion phenotype after carnosine intervention. Mitochondrial fission can separate damaged fragments [32], and fusion can repair damaged mitochondria or dilute damage by mixing mtDNA, proteins and lipids [33]. Therefore, it is suggested that carnosine may regulate the dynamic balance of mitochondrial fission and fusion, and help skeletal muscle cells adapt to environmental changes.

Slow muscle fibers are rich in mitochondria, and enhanced mitochondrial biosynthesis can promote the transformation of fast muscle fibers into slow muscle fibers and improve skeletal muscle quality and function. On the contrary, the decrease in mitochondrial biosynthesis ability is an important reason for the loss of skeletal muscle mass and functional decline [34]. Therefore, in addition to detecting ATP6 distribution in in vivo experiments, this study also focused on skeletal muscle fiber-type conversion, which is a core factor in maintaining metabolic homeostasis and muscle function, and is closely related to mitochondrial dysfunction and the progression of T2DM sarcopenia. In T2DM sarcopenia mice, the transformation of slow muscle fibers (oxidative) to fast muscle fibers (glycolytic) in skeletal muscle is an important factor in inducing insulin resistance and skeletal muscle mass loss [35]. The results of Western blotting in vivo showed that carnosine intervention improved skeletal muscle atrophy in T2DM sarcopenic mice (Figure 8A,B). At the same time, the results of immunofluorescence staining showed that the proportion of slow muscle fibers in gastrocnemius muscle increased after carnosine intervention (Figure 8D), suggesting that carnosine may affect the type of muscle fibers by promoting mitochondrial biosynthesis and improving mitochondrial metabolism efficiency, thereby improving skeletal muscle atrophy and dysfunction caused by T2DM.

Beyond the PI3K/AMPK/PGC-1α-mediated mitochondrial biogenesis and fiber-type switching, carnosine has additional activities that may protect against diabetic sarcopenia. It acts as an antioxidant (scavenging ROS, upregulating SOD/CAT/GPx [36]), an anti-inflammatory agent (inhibiting NF-κB, reducing TNF-α/IL-6, promoting M1 to M2 polarization [37]), and an anti-glycation agent (competing with sugars for protein binding, limiting AGEs [38]). Given that oxidative stress, inflammation, and AGEs are key features of T2DM sarcopenia, these mechanisms likely synergize with the PI3K/AMPK/PGC-1α pathway. Although not directly tested, their potential contribution warrants further investigation.

In this study, the molecular mechanism of carnosine in alleviating T2DM sarcopenia in mice was innovatively revealed through multi-omics combined in vitro and in vivo experiments, but there were some limitations. First, in order to eliminate the interference of hormones, only male mice were used for modeling, while there were gender differences in the pathogenesis of T2DM sarcopenia. The intervention effect and clinical applicability of carnosine on female patients need to be verified by gender stratification experiments and clinical studies. The second is the lack of clinical research on T2DM sarcopenia. The results of this study are limited to basic experiments, and follow-up animal and clinical studies with large samples and long-term follow-up are needed to quantify the effect of carnosine on skeletal muscle mass, muscle strength and motor function, and provide a basis for clinical transformation. Thirdly, we note that carnosine is known to possess pH buffering capacity; however, this study did not include separate control experiments (e.g., using TAE) to distinguish its pH-modulating effects from its signaling actions. It should be noted that the culture medium used in our experiments already contained HEPES, which itself helps maintain pH stability. If buffering were the primary mechanism, other buffering agents might also produce similar effects, but current literature lacks evidence to support this. Future studies that incorporate appropriate pH buffering controls would help further clarify its mechanism of action. Fourth, the metabolic characterization of our T2DM sarcopenia model was primarily limited to fasting blood glucose, body weight, and skeletal muscle atrophy parameters. We did not systematically evaluate other metabolic features characterizing the T2DM state, such as serum insulin levels or insulin resistance indices (e.g., HOMA-IR). Consequently, whether carnosine supplementation affects these additional metabolic parameters remains to be investigated in future studies.

In addition, it is necessary to point out the limitations of this study regarding mechanistic validation. Although molecular docking results showed that carnosine has a high binding energy with the PI3K p110β subunit, and inhibitor experiments demonstrated that PI3K activity is functionally required for the anti-atrophic effect of carnosine, these data only indicate an association between carnosine and the PI3K/AMPK/PGC-1α signaling pathway, and cannot prove that carnosine directly targets PI3K or physically binds to this protein. Molecular docking is merely a computational prediction; direct interaction between the two needs to be further validated by experimental approaches such as co-immunoprecipitation (Co-IP), surface plasmon resonance (SPR), or cellular thermal shift assay (CETSA). Therefore, the conclusion of this study should be interpreted as follows: exogenous carnosine supplementation is closely associated with the activation of the PI3K/AMPK/PGC-1α pathway and the alleviation of muscle atrophy, but its exact direct molecular target remains to be elucidated in future studies.

Despite the above limitations, this study still provides an important theoretical basis and potential targets for the intervention of T2DM sarcopenia. This study confirmed that it can improve the muscle atrophy phenotype of T2DM sarcopenia through the PI3K/AMPK/PGC-1α pathway, providing new ideas and an experimental basis for clinical intervention. With the deepening of research, it is expected to become a new auxiliary intervention for the disease.

## 5. Conclusions

In this study, a high-fat diet combined with STZ was used to successfully construct a T2DM sarcopenia mouse model. Metabolomics sequencing confirmed that T2DM could lead to a significant decrease in the content of carnosine in the skeletal muscle of mice. Both in vivo and in vitro experiments showed that high-glucose was a key factor in the metabolic disorder of carnosine metabolism. In vitro myotube experiments confirmed that carnosine could promote mitochondrial biosynthesis through the PI3K/AMPK/PGC-1α signaling pathway to alleviate high-glucose-induced myotube atrophy. In vivo experiments confirmed that carnosine may mediate the increase in slow muscle fiber ratio through this pathway, and ultimately improve skeletal muscle atrophy in T2DM sarcopenia mice.

## Data Availability

The LC-MS metabolomics sequencing and RNA-seq data generated in this study have been publicly deposited in the figshare repository and are accessible under the following DOIs: https://doi.org/10.6084/m9.figshare.32594154 and https://doi.org/10.6084/m9.figshare.32597670. Additional data that support the findings of this study are available from the corresponding author upon reasonable request.

## Supplementary Materials

The following supporting information can be downloaded at: https://www.mdpi.com/article/10.3390/biology15130999/s1, Figure S1: Analysis of gene expression saturation curves across various C2C12 myotubes samples. HG1, HG2 and HG3 represent myotubes treated with 10 mM glucose, while HG_CAR1, HG_CAR2, HG_CAR3 represent myotubes co-treated with 10 mM glucose and 8 mM Carnosine. Figure S2: SYBR Green quantitative RT-PCR validation of differentially expressed genes identified by transcriptome sequencing of carnosine-attenuated atrophy in C2C12 myotubes. n = 6, **** *p* < 0.0001, *** *p* < 0.001, ** *p* < 0.01. HG denotes treatment with 10 mM glucose. Figure S3. SYBR Green quantitative RT-PCR results of mtDNA copy number in C2C12 myotubes following carnosine treatment under high-glucose. RPP30 gene was used as a reference. HG denotes treatment with 10 mM glucose in this figure. n = 6 biological replicates. ns indicates no significant difference between groups, **** *p* < 0.0001, *** *p* < 0.001, ** *p* < 0.01. Figure S4. ATP content in C2C12 myotubes following carnosine treatment under high-glucose. HG denotes treatment with 10 mM glucose in this figure. n = 6 biological replicates. ns indicates no significant difference between groups, **** *p* < 0.0001, *** *p* < 0.001. Figure S5. ROS production in C2C12 myotubes following carnosine treatment under high-glucose. (A) ROS production staining images in C2C12 myotubes. (B) Statistical data of relative ROS fluorescence intensity. Blue fluorescence indicates DAPI stained nuclei, green fluorescence indicates ROS positive signals. HG denotes treatment with 10 mM glucose. n = 3 biological replicates. ns indicates no significant difference between groups; **** *p* < 0.0001. Table S1: Primer sequences for gene detection in this study; Table S2: Information of antibodies used in this study; Table S3: Overview of metabolome sequencing results; Table S4: Summary of sequencing data quality.
