# Supplementary material for: The Role and Mechanism of Carnosine in Alleviating Type 2 Diabetic Sarcopenia in Mice Through PI3K/AMPK/PGC-1α Signaling Pathway
Source: Biology (Basel). 2026 Jun 25;15(13):999. doi: 10.3390/biology15130999 (PMC13359430; doi:10.3390/biology15130999)
Supplement: Supplementary file 1 [file biology-15-00999-s001.zip › Supplementary Files/Figure S1.pdf]

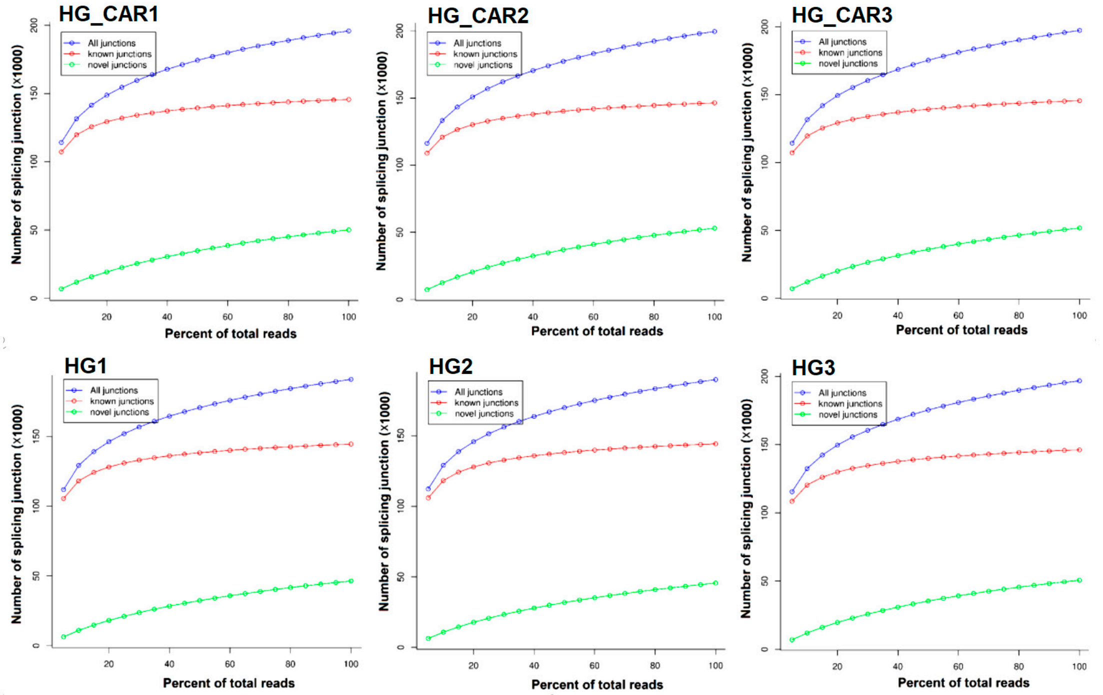

Supplementary Figure S1 Analysis of gene expression saturation curves across various C2C12 myotubes samples. HG1, HG2 and HG3 represent myotubes treated with 10 mM glucose, while HG_CAR1, HG_CAR2, HG_CAR3 represent myotubes co-treated with 10 mM glucose and 8 mM Carnosine.
