# Supplementary material for: The Role and Mechanism of Carnosine in Alleviating Type 2 Diabetic Sarcopenia in Mice Through PI3K/AMPK/PGC-1α Signaling Pathway
Source: Biology (Basel). 2026 Jun 25;15(13):999. doi: 10.3390/biology15130999 (PMC13359430; doi:10.3390/biology15130999)
Supplement: Supplementary file 1 [file biology-15-00999-s001.zip › Supplementary Files/Figure S3.pdf]

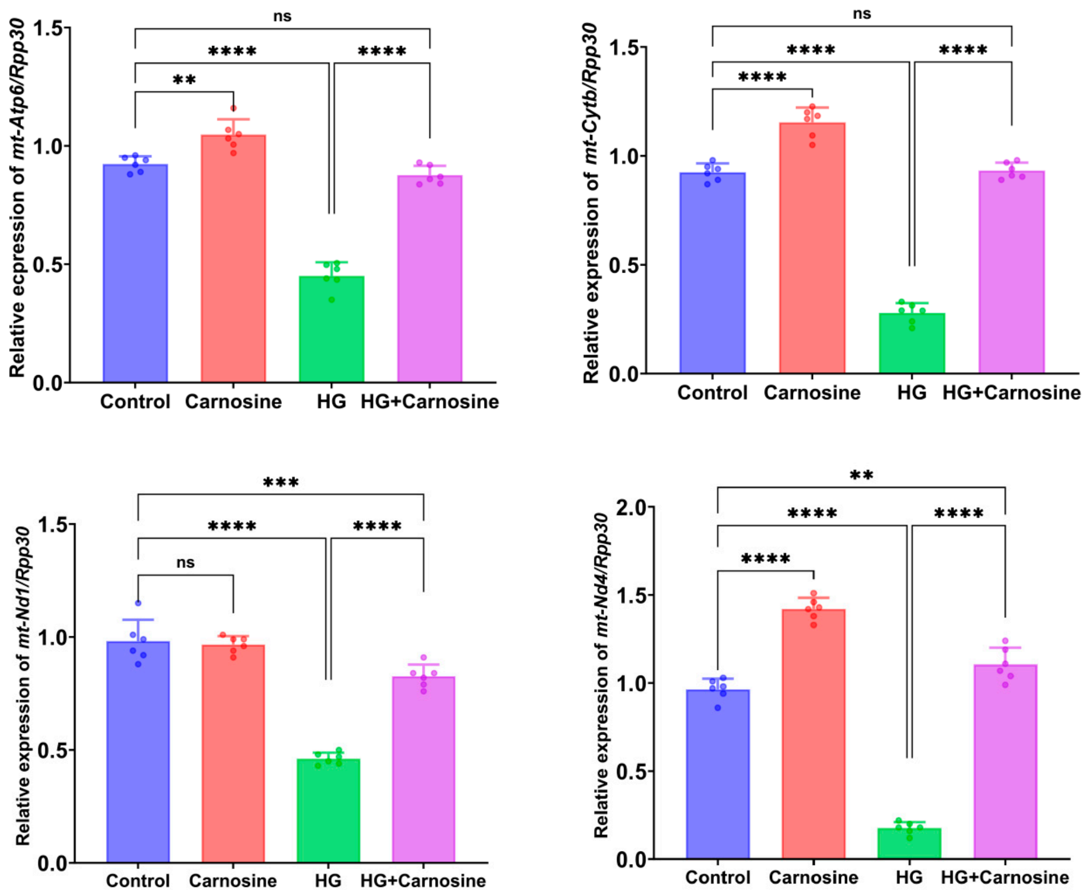

Supplementary Figure S3. SYBR Green quantitative RT-PCR results of mtDNA copy number in C2C12 myotubes following carnosine treatment under high glucose. *RPP30 gene was used as a reference*. HG denotes treatment with 10 mM glucose in this figure. n = 6 biological replicates. ns indicates no significant difference between groups, ****P <0.0001, ***P <0.001, **P <0.01.
