# Supplementary material for: The Role and Mechanism of Carnosine in Alleviating Type 2 Diabetic Sarcopenia in Mice Through PI3K/AMPK/PGC-1α Signaling Pathway
Source: Biology (Basel). 2026 Jun 25;15(13):999. doi: 10.3390/biology15130999 (PMC13359430; doi:10.3390/biology15130999)
Supplement: Supplementary file 1 [file biology-15-00999-s001.zip › Supplementary Files/Figure S5.pdf]

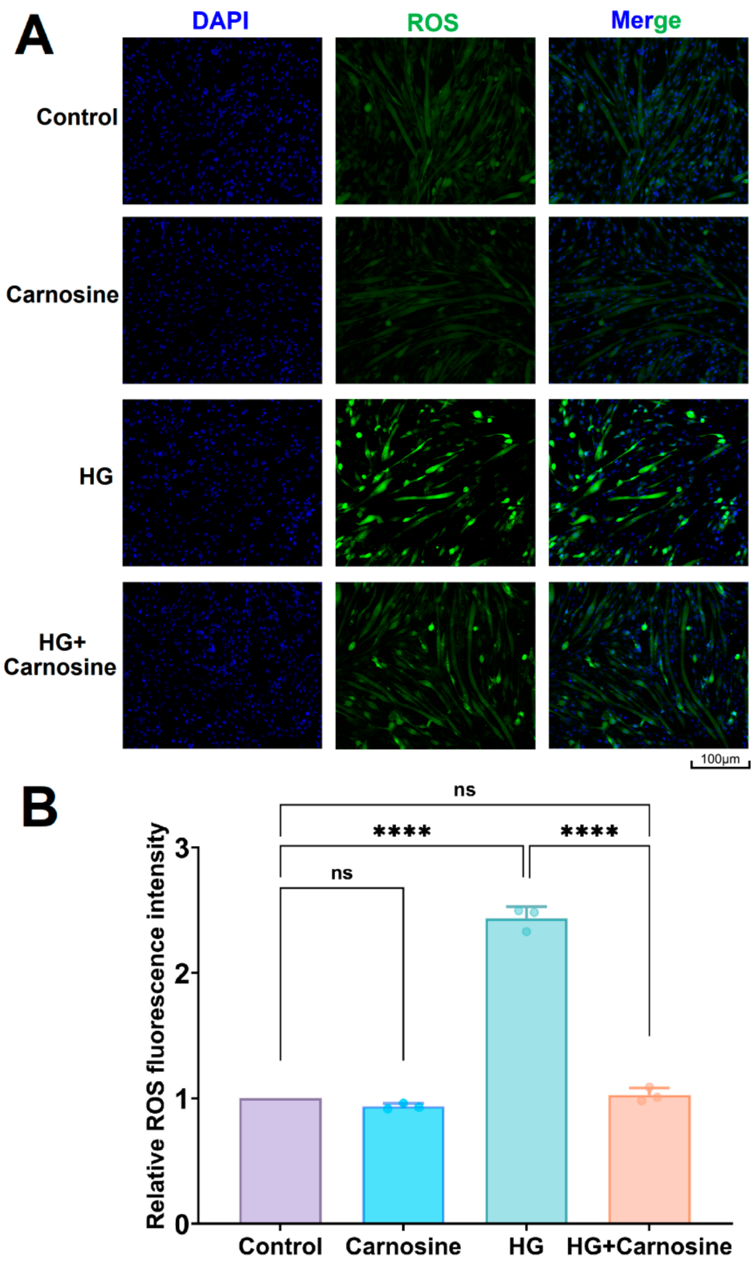

Supplementary Figure S5. ROS production in C2C12 myotubes following carnosine treatment under high glucose. (A) ROS production staining images in C2C12 myotubes. (B) Statistical data of relative ROS fluorescence intensity. Blue fluorescence indicates DAPI stained nuclei, green fluorescence indicates ROS positive signals. HG denotes treatment with 10 mM glucose. n = 3 biological replicates. ns indicates no significant difference between groups; ****P < 0.0001.
