# Supplementary material for: The Role and Mechanism of Carnosine in Alleviating Type 2 Diabetic Sarcopenia in Mice Through PI3K/AMPK/PGC-1α Signaling Pathway
Source: Biology (Basel). 2026 Jun 25;15(13):999. doi: 10.3390/biology15130999 (PMC13359430; doi:10.3390/biology15130999)
Supplement: Supplementary file 1 [file biology-15-00999-s001.zip › Supplementary Files/Table S1.pdf]

Supplementary Table S1 Primer sequences for gene detection in this study

Gene names	Sequences information
TFAM	*F:*TCGCATCCCCTCGTCTATCA; *R:* CCACAGGGCTGCAATTTTCC
TFB2M	*F:*GGCCCATCTTGCATTCTAGGG; *R:* GCAACGGCTCTATATTGAAGTCA
CARNS	*F:*TGATAGGCCCCTACTGAGTAAGGT; *R:* TCAGTGTCCTTGGCAGGGTAT
CNDP2	*F:*GGAGATACCACTTCCTCCCATTC; *R: F:*CGTCCAGGTGCCCGTAAAT
PEPT1	*F:*CTTGGAGCCACCACAATGG; *R:* ACAGAATTCATTGACCACGATGA
PHT1	*F:*CATGTGTCCGTGGTGATTGAG; *R:* GCGTGGTGTAACTGCCAATCT
PAT1	*F:*TCTGCTGTGTCTACTTCGTGTTTCT; *R:* GGATCACGGTCACATTGTTGTT
TauT	*F:*TGGCCGACAGCATTCCA; *R:* GCCTTCTCTAAGGTGCCTTCCT
Pik3c2g	*F:*AGGACTCGAGGCTCACCAT; *R:* TCCGAACTTCACTGGGATGTG
Atp1a4	*F:*TATTACCAGGAGGCCAAGAGC; *R:* GCGCCGAGATAACCCTGATA
Perm1	*F:*GTCGTAAGAAGAGGCGTGCT; *R:* GTGTCCCAGACATGAGAGCC
Wfikkn1	*F:*TGTCACCAGTATCGGACAGC; *R:* CCCTGTCCTGAGTAGTTGCC
mt-ATP6	*F:*GTCCTTTTGGTGTGTGGATTAGC; *R:* CCCATCCTCAAAACGCCTAA
mt-Cytb	*F:*AGGAGGTGTCCTAGCCTTAAT; *R:* AATTTGTGTGATTGGGCGGA
mt-Nd1	*F:*TCCGAGCATCTTATCCACGC; *R:* GTATGGTGGTACTCCCGCTG
mt-Nd4	*F:*AGCTCAATCTGCTTACGCCA; *R:* TGTGAGGCCATGTGCGATTA
Rpp30	*F:*TGACGTGGCAAACTTAGGACT; *R:* TGATGGCCGTGGTTTCTTCA
GAPDH	*F:* GACATGCCGCCTGGAGAAAC; *R:* AGCCCAGGATGCCCTTTAGT
