# Supplementary material for: The Role and Mechanism of Carnosine in Alleviating Type 2 Diabetic Sarcopenia in Mice Through PI3K/AMPK/PGC-1α Signaling Pathway
Source: Biology (Basel). 2026 Jun 25;15(13):999. doi: 10.3390/biology15130999 (PMC13359430; doi:10.3390/biology15130999)
Supplement: Supplementary file 1 [file biology-15-00999-s001.zip › Supplementary Files/Table S2.pdf]

Supplementary Table S2 Information of antibodies used in this study

Names	Catalog Numbers
Anti-PI3 Kinase p110 beta Rabbit (PI3K)	Bioss（bs-10657R）
Anti-phospho-PI3 Kinase p110 beta (Ser1070) Rabbit (p-PI3K)	Bioss（bs-6417R）
Anti-PGC-1α	Proteintech（66369-1-Ig）
Anti-ATP6	Beyotime（AF6261）
Anti-AMPK	Cell signaling technology（2532）
Anti-Atrogin-1	Proteintech（67172-1-Ig）
Anti-MuRF1	Proteintech（82345-4-RR）
Anti-MyoG	Proteintech（55069-1-AP）
Anti-Desmin	Bioss（bsm-60266R）
Anti-beta-Tubulin	Bioss（bsm-33034R
Goat-anti-Rabbit-FITC	Bioss（bs-0295G-FITC）
Goat-anti-Rabbit-RBITC	Bioss（bs-0295G-RBITC）
Goat-anti-Rabbit-BF647	Bioss（bs-0293G-BF647）
Anti-Carnosine	Cosmo Bio（GEM-AP055）
