# Supplementary material for: The Role and Mechanism of Carnosine in Alleviating Type 2 Diabetic Sarcopenia in Mice Through PI3K/AMPK/PGC-1α Signaling Pathway
Source: Biology (Basel). 2026 Jun 25;15(13):999. doi: 10.3390/biology15130999 (PMC13359430; doi:10.3390/biology15130999)
Supplement: Supplementary file 1 [file biology-15-00999-s001.zip › Supplementary Files/Table S3.pdf]

Supplementary Table S3 Overview of metabolome sequencing results

Ion mode	Peaks	Identified metabolites	Metabolites in Library	Metabolites in KEGG
Positive ion mode	2364	486	428	247
Negative ion mode	2896	489	473	287
Mixed mode	5260	975	901	534
