# Supplementary material for: The Role and Mechanism of Carnosine in Alleviating Type 2 Diabetic Sarcopenia in Mice Through PI3K/AMPK/PGC-1α Signaling Pathway
Source: Biology (Basel). 2026 Jun 25;15(13):999. doi: 10.3390/biology15130999 (PMC13359430; doi:10.3390/biology15130999)
Supplement: Supplementary file 1 [file biology-15-00999-s001.zip › Supplementary Files/Table S4.pdf]

Supplementary Table S4 Summary of sequencing data quality

Sample name	Raw reads	Clean reads	Raw base	Clean base	Error rate(%)	Q20(%)	Q30(%)	GC content(%)
HG1	45797118	43795504	6.75	6.49	0.01	99.49	97.21	50.2
HG2	45005530	42705554	6.6	6.32	0.01	99.53	97.21	50.5
HG3	48573404	46289922	7.29	7.38	0.01	99.46	97.02	50.05
HG_CAR1	46655092	54010202	7.5	7.11	0.01	99.53	97.34	50.11
HG_CAR2	55520502	54695536	7.7	6.35	0.01	99.49	97.16	49.98
HG_CAR3	46370496	44083560	6.96	6.78	0.01	99.48	97.16	49.06

Note: HG1, HG2 and HG3 represent myotubes treated with 10 mM glucose, while HG_CAR1, HG_CAR2, HG_CAR3 represent myotubes co-treated with 10 mM glucose and 8 mM Carnosine.
