# Supplementary figures and images for: The Role and Mechanism of Carnosine in Alleviating Type 2 Diabetic Sarcopenia in Mice Through PI3K/AMPK/PGC-1α Signaling Pathway
Source: Biology (Basel). 2026 Jun 25;15(13):999. doi: 10.3390/biology15130999 (PMC13359430; doi:10.3390/biology15130999)

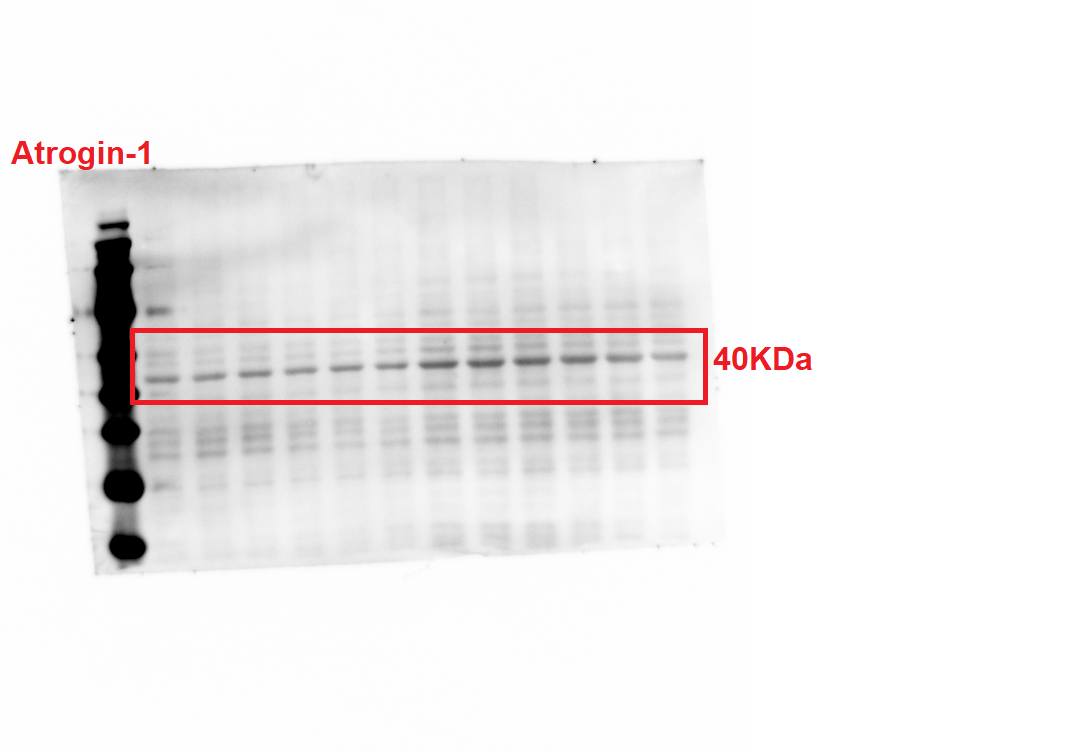

Supplement: Supplementary file 1 [file biology-15-00999-s001.zip › Original data of WB/Figure 3/Atrogin.tif]

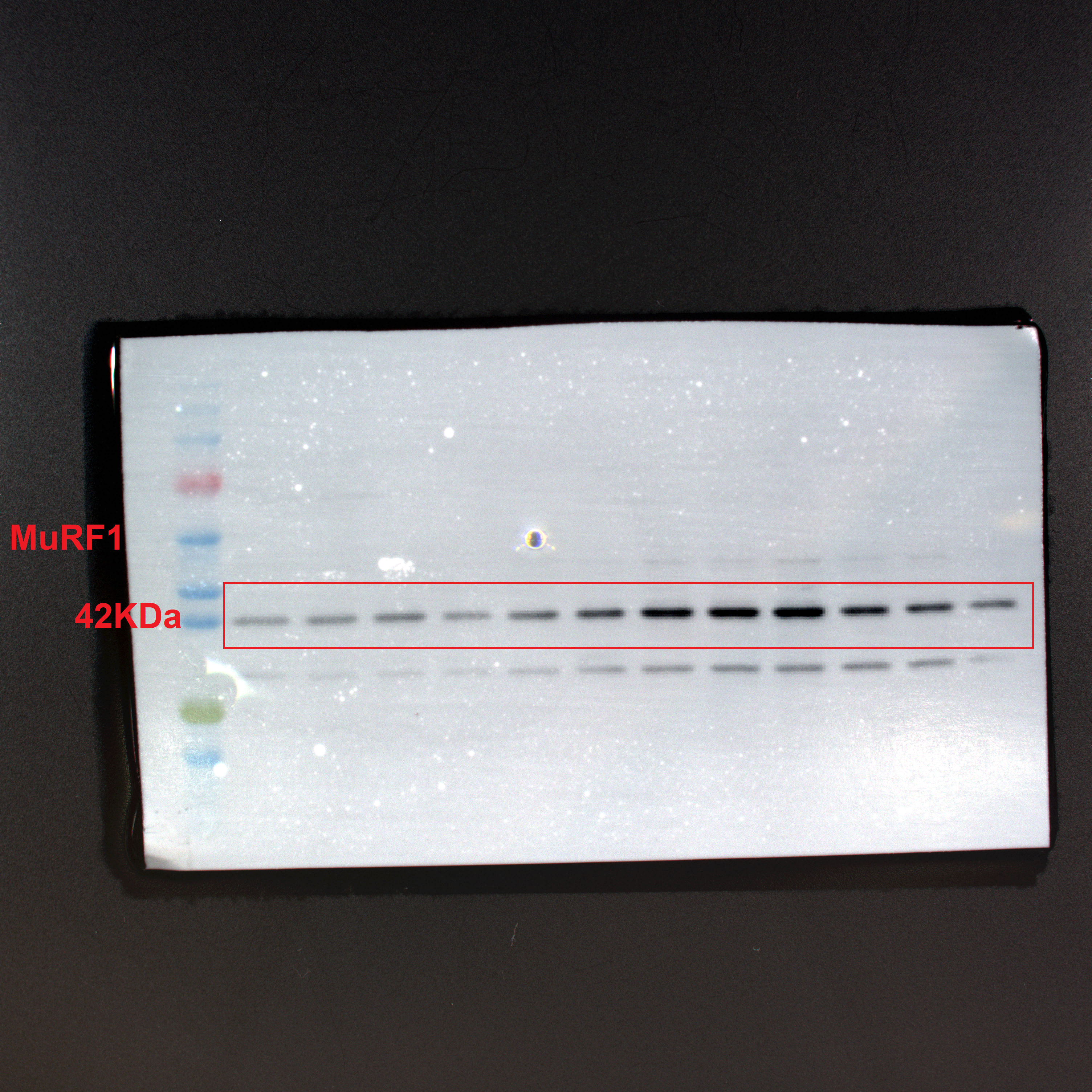

Supplement: Supplementary file 1 [file biology-15-00999-s001.zip › Original data of WB/Figure 3/MuRF.tif]

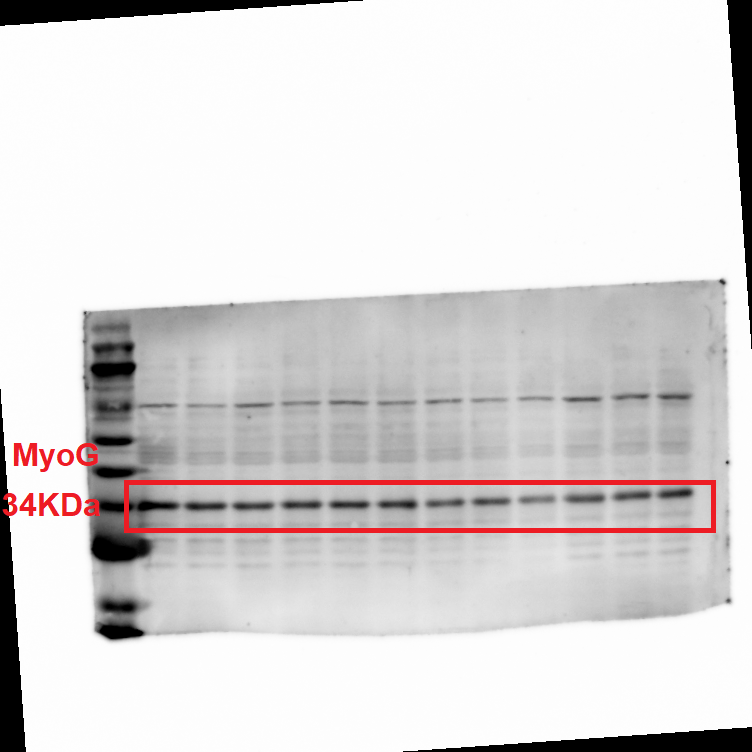

Supplement: Supplementary file 1 [file biology-15-00999-s001.zip › Original data of WB/Figure 3/myog.tif]

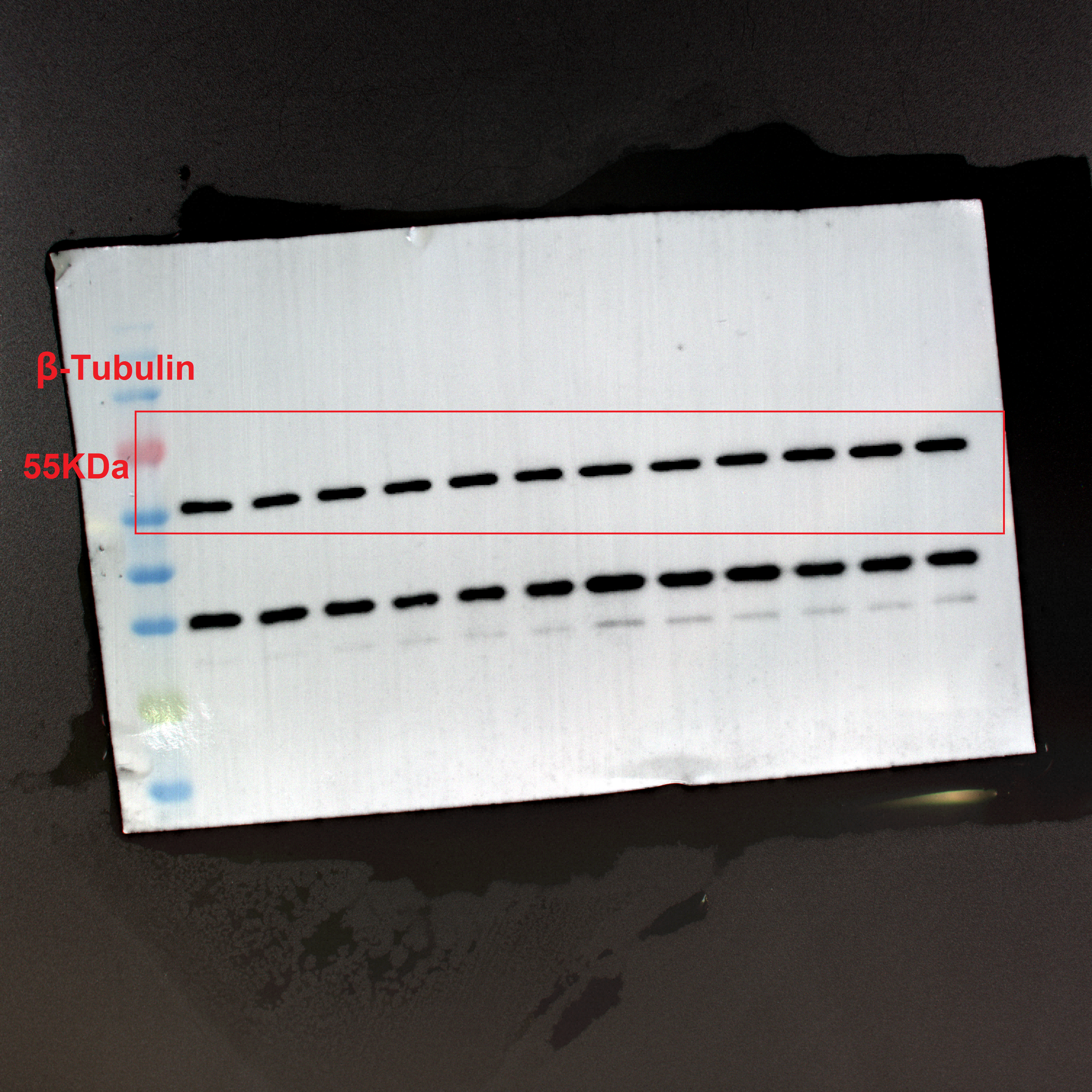

Supplement: Supplementary file 1 [file biology-15-00999-s001.zip › Original data of WB/Figure 3/Tubulin.tif]

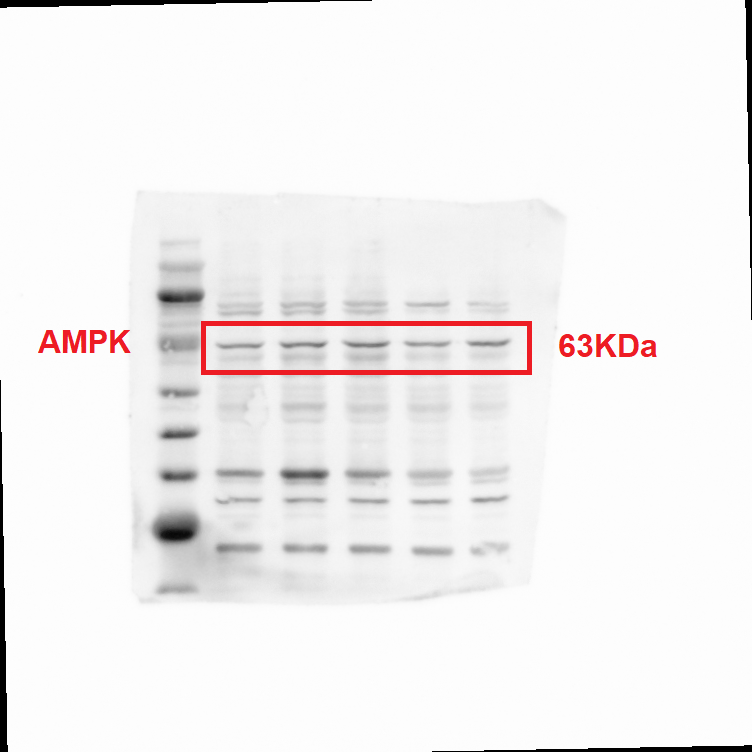

Supplement: Supplementary file 1 [file biology-15-00999-s001.zip › Original data of WB/Figure 6/AMPK.tif]

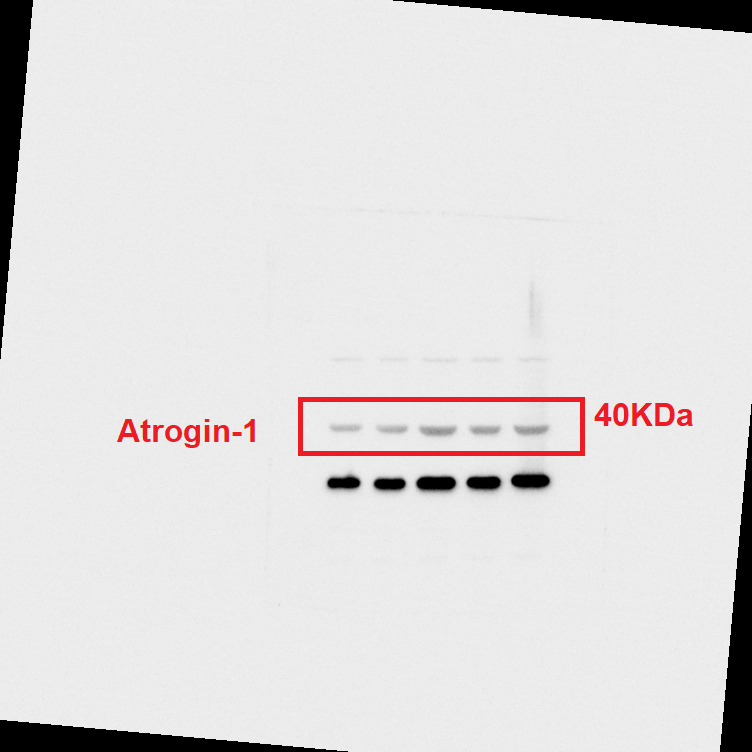

Supplement: Supplementary file 1 [file biology-15-00999-s001.zip › Original data of WB/Figure 6/Atrogin-1.tif]

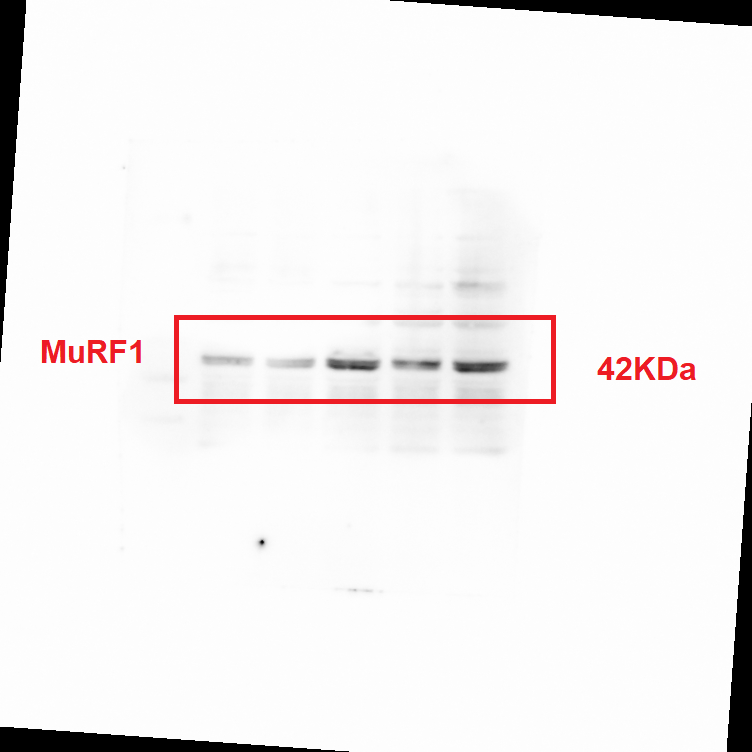

Supplement: Supplementary file 1 [file biology-15-00999-s001.zip › Original data of WB/Figure 6/Murf1.tif]

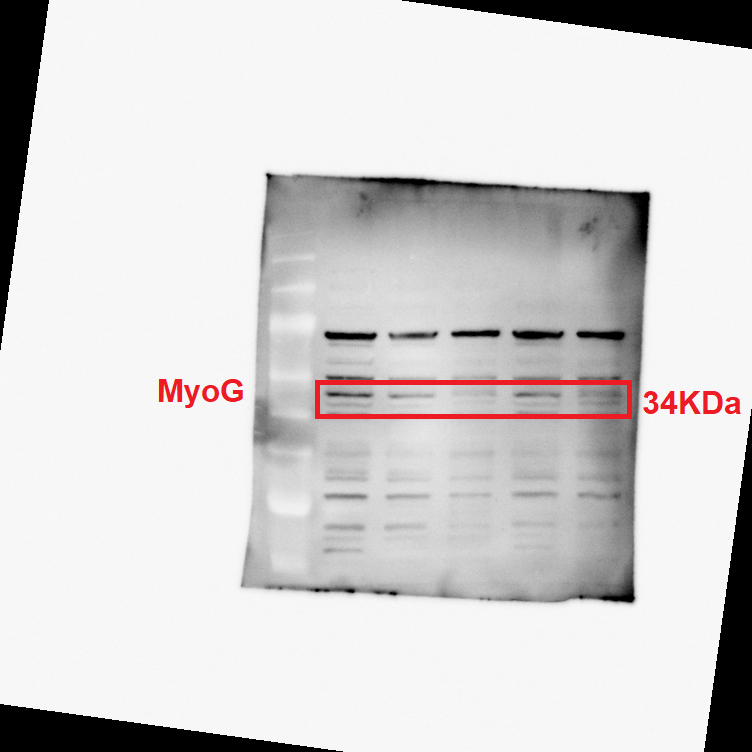

Supplement: Supplementary file 1 [file biology-15-00999-s001.zip › Original data of WB/Figure 6/MyoG.tif]

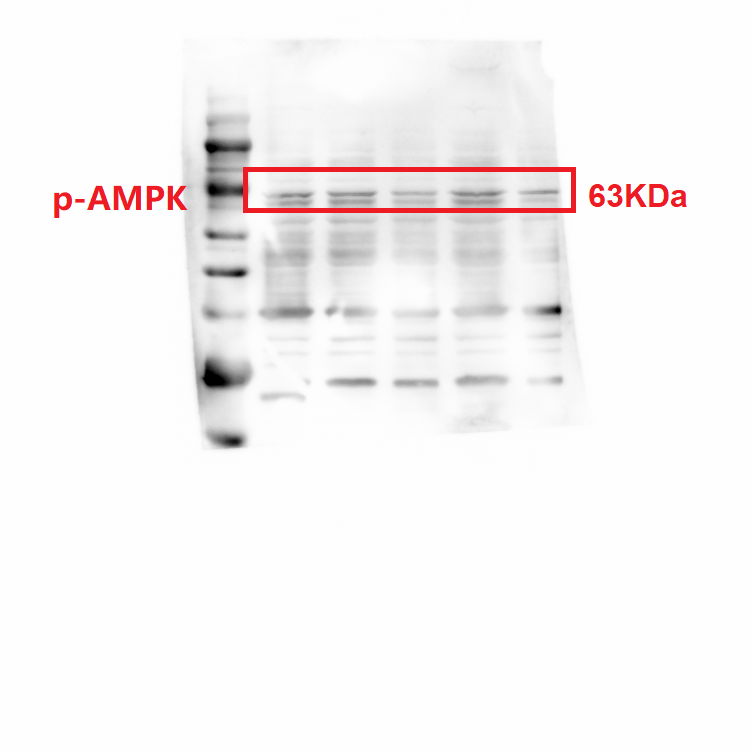

Supplement: Supplementary file 1 [file biology-15-00999-s001.zip › Original data of WB/Figure 6/p-ampk.tif]

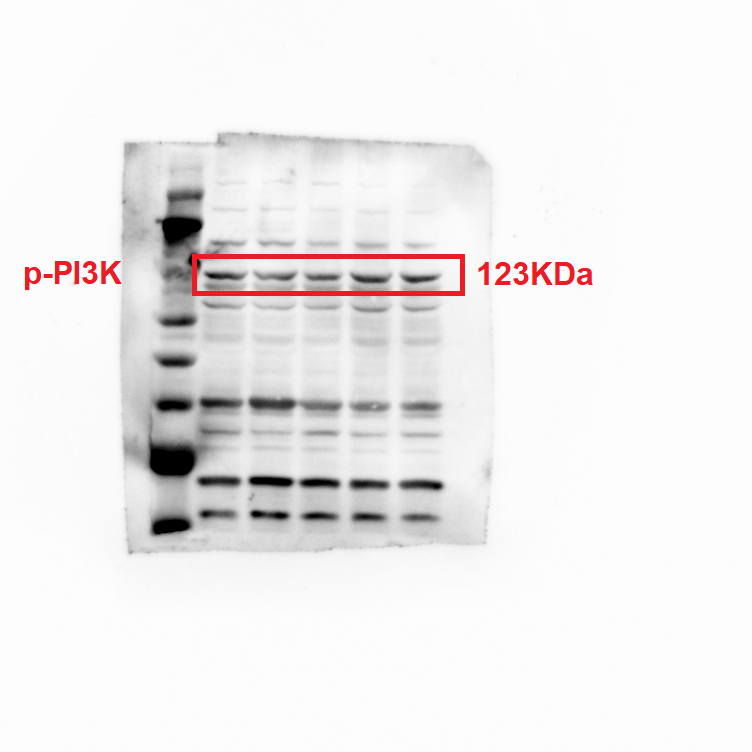

Supplement: Supplementary file 1 [file biology-15-00999-s001.zip › Original data of WB/Figure 6/p-PI3K.tif]

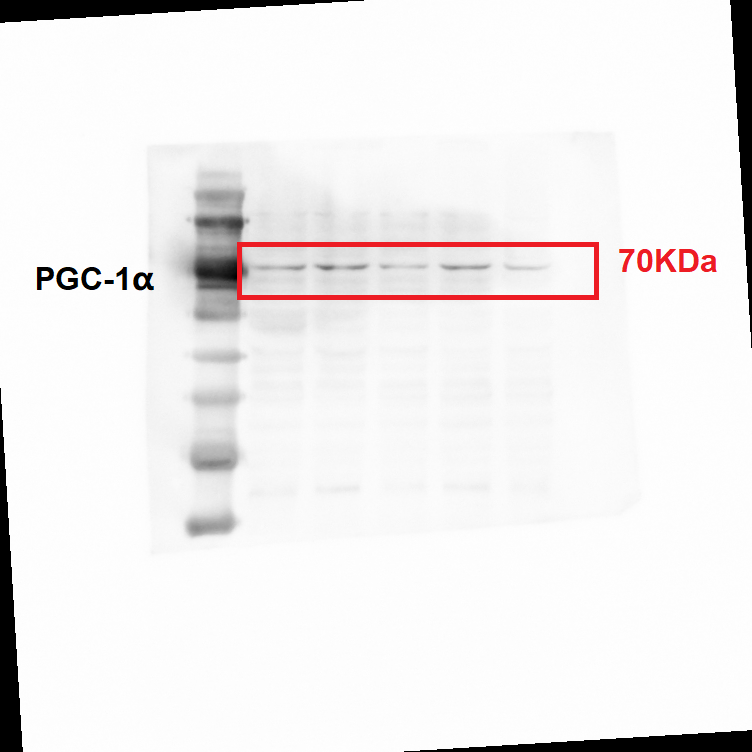

Supplement: Supplementary file 1 [file biology-15-00999-s001.zip › Original data of WB/Figure 6/pgc1α.tif]

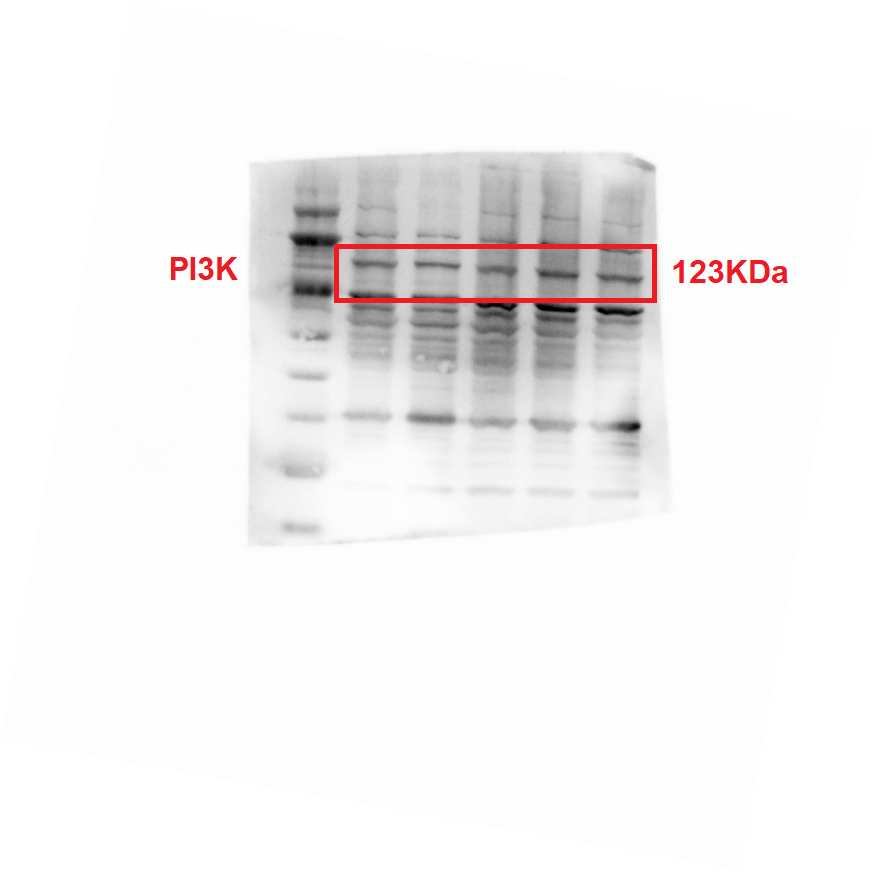

Supplement: Supplementary file 1 [file biology-15-00999-s001.zip › Original data of WB/Figure 6/PI3K.tif]

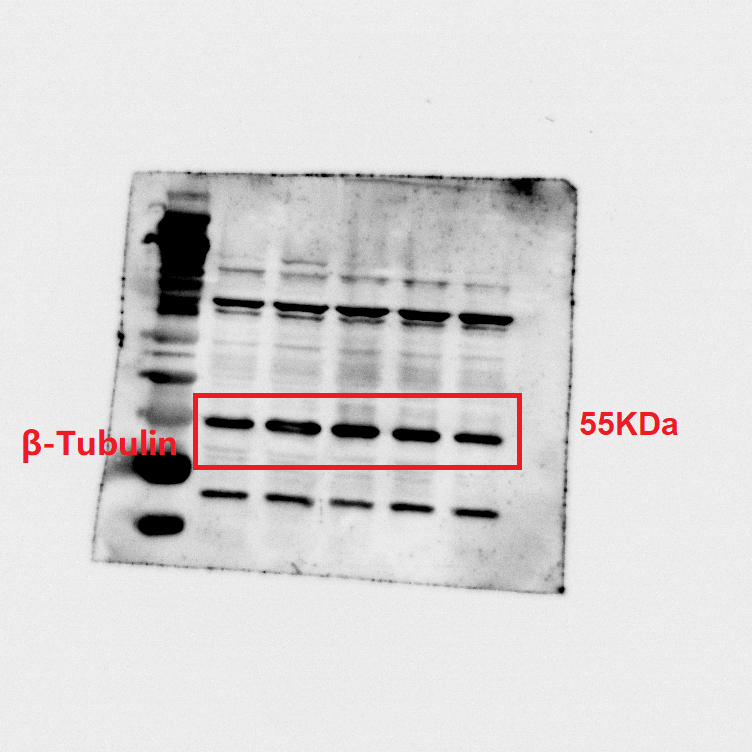

Supplement: Supplementary file 1 [file biology-15-00999-s001.zip › Original data of WB/Figure 6/Tubulin.tif]

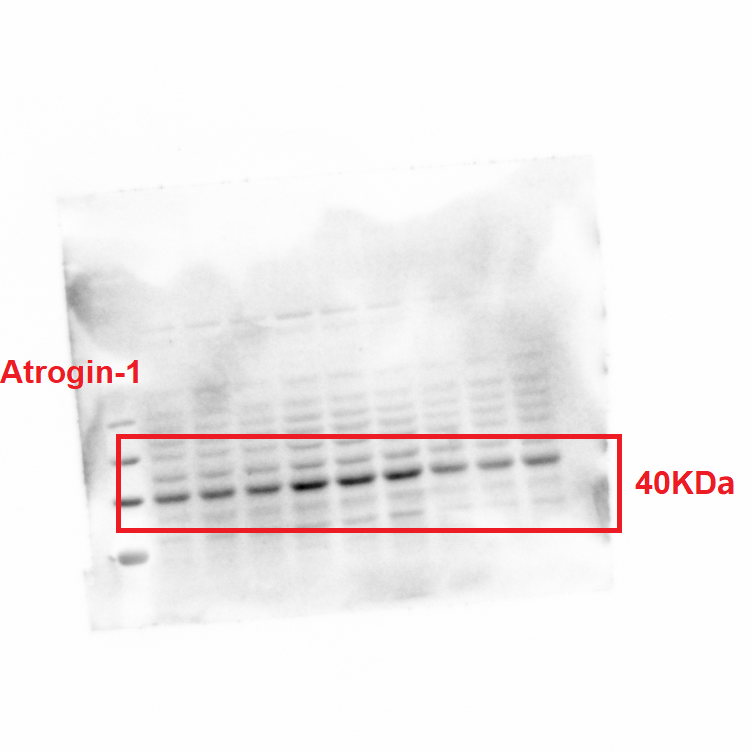

Supplement: Supplementary file 1 [file biology-15-00999-s001.zip › Original data of WB/Figure 8/Atrogin-1.tif]

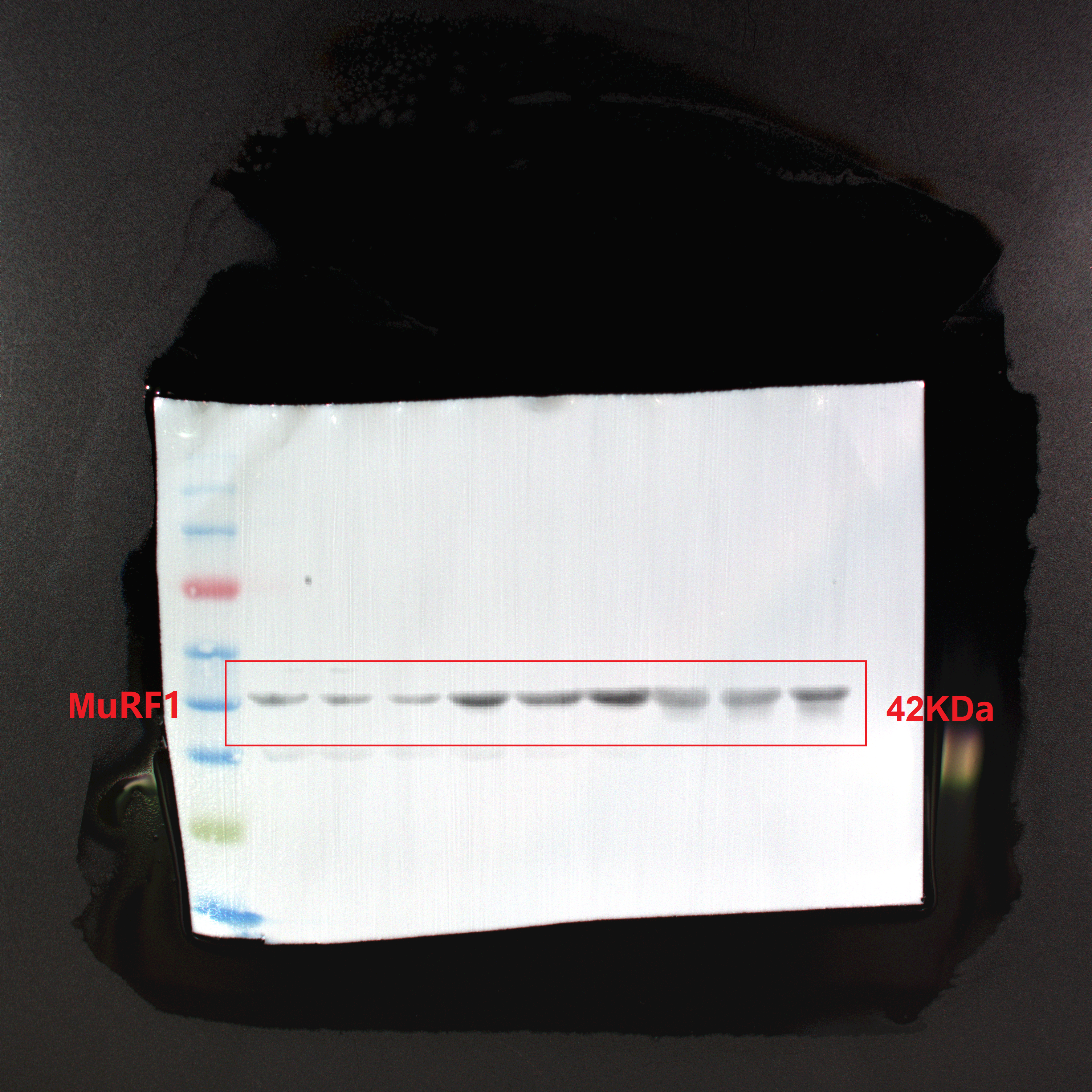

Supplement: Supplementary file 1 [file biology-15-00999-s001.zip › Original data of WB/Figure 8/MuRF1.tif]

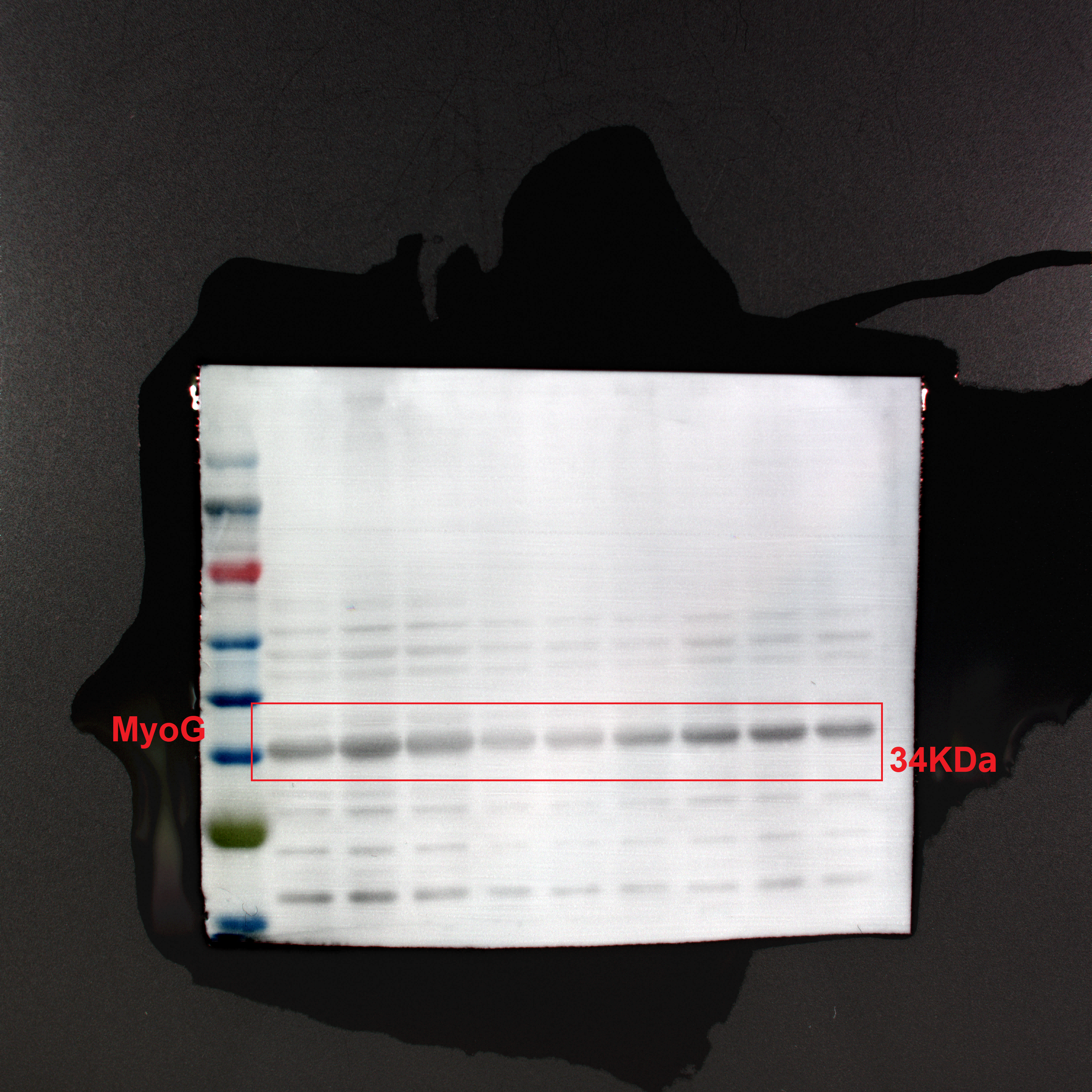

Supplement: Supplementary file 1 [file biology-15-00999-s001.zip › Original data of WB/Figure 8/myog.tif]

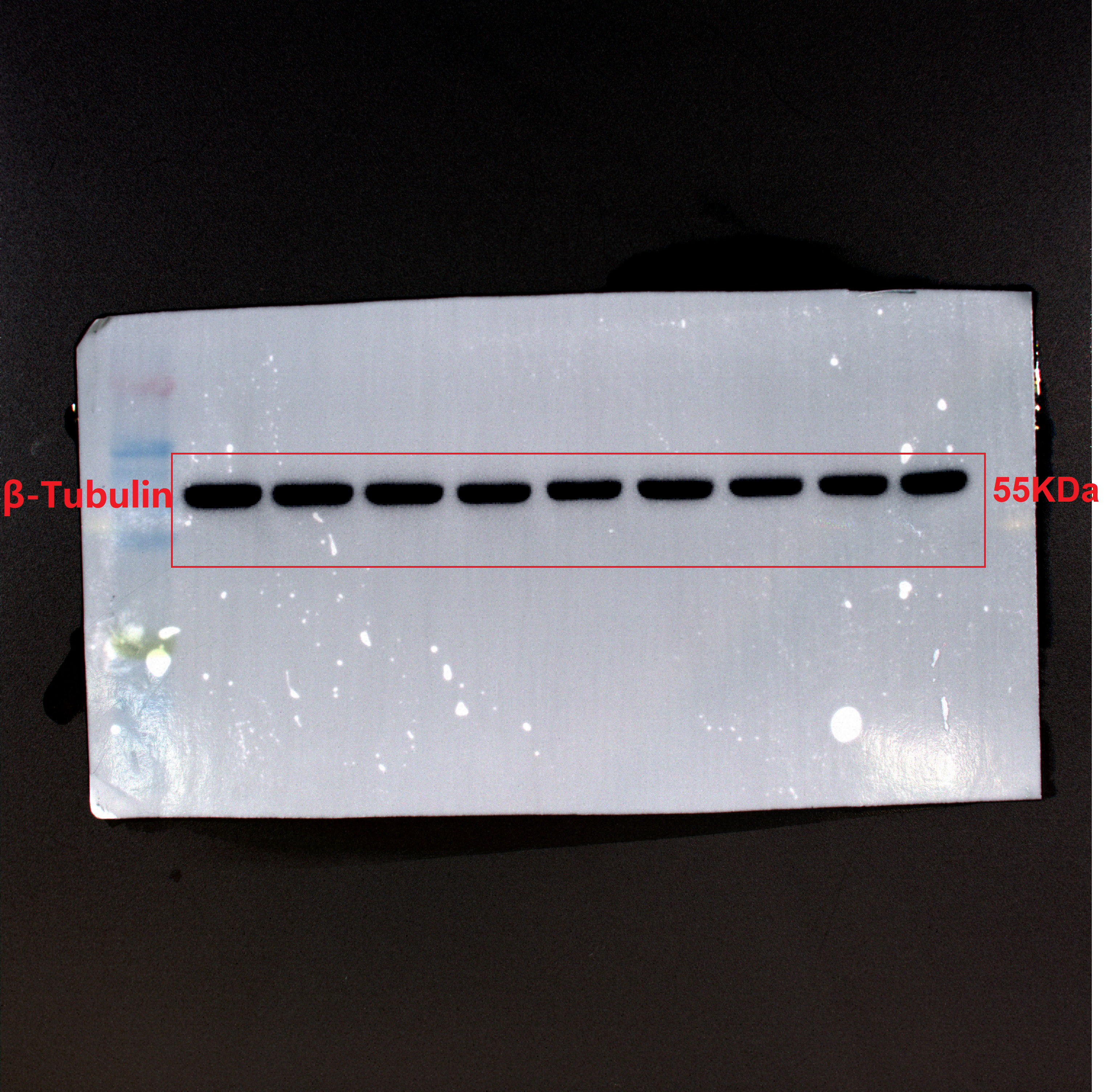

Supplement: Supplementary file 1 [file biology-15-00999-s001.zip › Original data of WB/Figure 8/Tubulin.tif]
